# Aging hallmarks of the primate ovary revealed by spatiotemporal transcriptomics

**DOI:** 10.1093/procel/pwad063

**Published:** 2023-12-21

**Authors:** Huifen Lu, Ying Jing, Chen Zhang, Shuai Ma, Weiqi Zhang, Daoyuan Huang, Bin Zhang, Yuesheng Zuo, Yingying Qin, Guang-Hui Liu, Yang Yu, Jing Qu, Si Wang

**Affiliations:** Advanced Innovation Center for Human Brain Protection, National Clinical Research Center for Geriatric Disorders, Xuanwu Hospital Capital Medical University, Beijing 100053, China; Aging Translational Medicine Center, International Center for Aging and Cancer, Beijing Municipal Geriatric Medical Research Center, Xuanwu Hospital, Capital Medical University, Beijing 100053, China; Advanced Innovation Center for Human Brain Protection, National Clinical Research Center for Geriatric Disorders, Xuanwu Hospital Capital Medical University, Beijing 100053, China; Aging Translational Medicine Center, International Center for Aging and Cancer, Beijing Municipal Geriatric Medical Research Center, Xuanwu Hospital, Capital Medical University, Beijing 100053, China; The Fifth People’s Hospital of Chongqing, Chongqing 400062, China; State Key Laboratory of Membrane Biology, Institute of Zoology, Chinese Academy of Sciences, Beijing 100101, China; Beijing Institute for Stem Cell and Regenerative Medicine, Beijing 100101, China; Institute for Stem cell and Regeneration, CAS, Beijing 100101, China; Key Laboratory of Organ Regeneration and Reconstruction, Institute of Zoology, Chinese Academy of Sciences, Beijing 100101, China; Aging Biomarker Consortium, Beijing 100101, China; University of Chinese Academy of Sciences, Beijing 100049, China; CAS Key Laboratory of Genomic and Precision Medicine, Beijing Institute of Genomics, Chinese Academy of Sciences, Beijing 100101, China; Institute for Stem cell and Regeneration, CAS, Beijing 100101, China; China National Center for Bioinformation, Beijing 100101, China; Sino-Danish College, University of Chinese Academy of Sciences, Beijing 101408, China; Sino-Danish Center for Education and Research, Beijing 101408, China; Aging Biomarker Consortium, Beijing 100101, China; Advanced Innovation Center for Human Brain Protection, National Clinical Research Center for Geriatric Disorders, Xuanwu Hospital Capital Medical University, Beijing 100053, China; Aging Translational Medicine Center, International Center for Aging and Cancer, Beijing Municipal Geriatric Medical Research Center, Xuanwu Hospital, Capital Medical University, Beijing 100053, China; State Key Laboratory of Membrane Biology, Institute of Zoology, Chinese Academy of Sciences, Beijing 100101, China; University of Chinese Academy of Sciences, Beijing 100049, China; Key Laboratory of Organ Regeneration and Reconstruction, Institute of Zoology, Chinese Academy of Sciences, Beijing 100101, China; University of Chinese Academy of Sciences, Beijing 100049, China; CAS Key Laboratory of Genomic and Precision Medicine, Beijing Institute of Genomics, Chinese Academy of Sciences, Beijing 100101, China; China National Center for Bioinformation, Beijing 100101, China; Center for Reproductive Medicine, Cheeloo College of Medicine, Shandong University, Jinan 250012, China; Key Laboratory of Reproductive Endocrinology of Ministry of Education, National Research Center for Assisted Reproductive Technology and Reproductive Genetics, Shandong Key Laboratory of Reproductive Medicine, Shandong Provincial Clinical Research Center for Reproductive Health, Jinan 250012, China; Advanced Innovation Center for Human Brain Protection, National Clinical Research Center for Geriatric Disorders, Xuanwu Hospital Capital Medical University, Beijing 100053, China; Aging Translational Medicine Center, International Center for Aging and Cancer, Beijing Municipal Geriatric Medical Research Center, Xuanwu Hospital, Capital Medical University, Beijing 100053, China; State Key Laboratory of Membrane Biology, Institute of Zoology, Chinese Academy of Sciences, Beijing 100101, China; University of Chinese Academy of Sciences, Beijing 100049, China; Beijing Institute for Stem Cell and Regenerative Medicine, Beijing 100101, China; Institute for Stem cell and Regeneration, CAS, Beijing 100101, China; Key Laboratory of Organ Regeneration and Reconstruction, Institute of Zoology, Chinese Academy of Sciences, Beijing 100101, China; Aging Biomarker Consortium, Beijing 100101, China; Department of Obstetrics and Gynecology, Center for Reproductive Medicine, Peking University, Third Hospital, Beijing 100191, China; Clinical Stem Cell Research Center, Peking University, Third Hospital, Beijing 100191, China; State Key Laboratory of Stem Cell and Reproductive Biology, Institute of Zoology, Chinese Academy of Sciences, Beijing 100101, China; University of Chinese Academy of Sciences, Beijing 100049, China; Beijing Institute for Stem Cell and Regenerative Medicine, Beijing 100101, China; Institute for Stem cell and Regeneration, CAS, Beijing 100101, China; Key Laboratory of Organ Regeneration and Reconstruction, Institute of Zoology, Chinese Academy of Sciences, Beijing 100101, China; Aging Biomarker Consortium, Beijing 100101, China; Advanced Innovation Center for Human Brain Protection, National Clinical Research Center for Geriatric Disorders, Xuanwu Hospital Capital Medical University, Beijing 100053, China; Aging Translational Medicine Center, International Center for Aging and Cancer, Beijing Municipal Geriatric Medical Research Center, Xuanwu Hospital, Capital Medical University, Beijing 100053, China; The Fifth People’s Hospital of Chongqing, Chongqing 400062, China; Aging Biomarker Consortium, Beijing 100101, China

**Keywords:** spatial transcriptome, primate, ovary, aging, senescence, inflammation

## Abstract

The ovary is indispensable for female reproduction, and its age-dependent functional decline is the primary cause of infertility. However, the molecular basis of ovarian aging in higher vertebrates remains poorly understood. Herein, we apply spatiotemporal transcriptomics to benchmark architecture organization as well as cellular and molecular determinants in young primate ovaries and compare these to aged primate ovaries. From a global view, somatic cells within the non-follicle region undergo more pronounced transcriptional fluctuation relative to those in the follicle region, likely constituting a hostile microenvironment that facilitates ovarian aging. Further, we uncovered that inflammation, the senescent-associated secretory phenotype, senescence, and fibrosis are the likely primary contributors to ovarian aging (PCOA). Of note, we identified spatial co-localization between a PCOA-featured spot and an unappreciated *MT2* (Metallothionein 2) highly expressing spot (MT2^high^) characterized by high levels of inflammation, potentially serving as an aging hotspot in the primate ovary. Moreover, with advanced age, a subpopulation of MT2^high^ accumulates, likely disseminating and amplifying the senescent signal outward. Our study establishes the first primate spatiotemporal transcriptomic atlas, advancing our understanding of mechanistic determinants underpinning primate ovarian aging and unraveling potential biomarkers and therapeutic targets for aging and age-associated human ovarian disorders.

## Introduction

As an important component of the female reproductive system, the ovary generates oocytes and is the major provider of steroid sex hormones (Adashi, 1994). Several cohort studies demonstrate that female fertility starts to decline around the late 20s to early 30s and declines more rapidly after the age of 35 (Zhu et al., 2022). Thus, ovarian aging is the primary cause of age-associated infertility in women. In addition, age-associated ovarian dysfunction often leads to endocrine imbalance (Vollenhoven and Hunt, 2018), which manifests as an irregular menstrual cycle and eventually menopause, the end of ovarian function but also, in and of itself, increases the risk of other disorders such as cardiovascular diseases, neurodegenerative diseases, and type 2 diabetes (Perry et al., 2015). Since ovarian aging precedes that of any other organ and tissue in women (Wu et al., 2022) and initiates a cascade of detrimental health effects, investigating ovarian aging in depth is both important and urgent.

The major characterization of ovarian aging is massive reductions in the quantity and quality of oocytes (Ahmed et al., 2020; Broekmans et al., 2009; Wang et al., 2020; Zhu et al., 2022). In addition, aging-associated hostile ovarian microenvironment that comprises ovarian somatic cells including granulosa cells, impairs follicle growth and maturation and serves as a primary confounding factor to ovarian functional decline, further leading to subfertility even infertility with advancing age (Ahmed et al., 2020). Therefore, it is imperative to delve into the cell type-specific alterations underlying ovarian aging. The application of emerging single-cell sequencing technologies has advanced our understanding of transcriptional profiles of various tissues and organs at diverse physiological and pathological conditions and at a high resolution and high accuracy (Cheng et al., 2021; He et al., 2020a, 2020b; Huang et al., 2022; Jing et al., 2023; Lehallier et al., 2019; Leng and Pawelec, 2022; Ma et al., 2023; Sun et al., 2023; Tang et al., 2009; Zhou et al., 2022). Taking advantage of single-cell transcriptomics, pioneering studies have revealed the cell compositions, and cell type-specific transcriptional features of ovarian development and aging in both rodents and primates (Isola et al., 2023; Jia et al., 2023; Lengyel et al., 2022; Wagner et al., 2020; Wang et al., 2020). However, due to high cell heterogeneity of the ovary and its intricate geographical distribution (Fan and Chuva de Sousa Lopes, 2021), progress toward understanding the cellular and molecular programs underlying ovarian degeneration from a spatial perspective, and particularly in primates, has remained limited.

Here, we established an in-depth spatial transcriptomic atlas of non-human primate (NHP) ovarian aging. Our atlas enabled us to resolve cellular and molecular alterations associated with ovarian aging at spatial resolution. Particularly, we identified that MT2^high^ spot characterized by prominent inflammation levels may serve as the senescence foci in the NHP ovary, which may augment senescence outward by secreting inflammatory factors. Collectively, through profiling the spatiotemporal landscape of primate ovarian aging, we provide a rich resource for identifying cellular and molecular programs that can be applied to evaluate ovarian aging and develop interventions against human ovarian aging and related diseases.

## Results

### Construction of a spatiotemporal transcriptomic atlas of NHP ovarian aging

**Figure 1.**
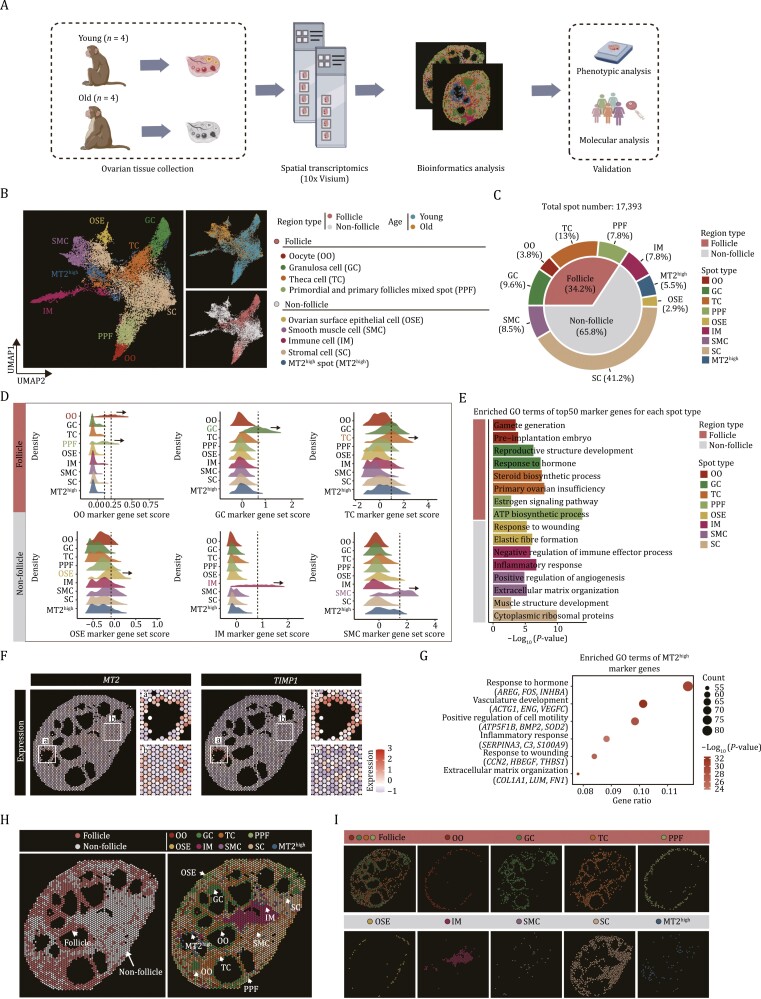
Construction of a spatial transcriptomic atlas of cynomolgus monkey ovarian aging. (A) Workflow showing the establishment of spatial transcriptomic atlas of young (*n* = 4) and old monkey (*n* = 4) ovaries and validations of changes of core factors associated with aging. (B) Uniform manifold approximation and projection (UMAP) plot showing the nine spot types from follicular and non-follicular regions and two age groups. (C) Pie chart showing the proportion of different spot types and region types of monkey ovary. (D) Ridge plots showing scores of known marker genes for multiple major ovarian spot types. (E) Pathway enrichment analysis for the top 50 marker genes of each spot type. (F) Spatial expression of two marker genes (*MT2* and *TIMP1*) for MT2^high^ spot represented by a young representative individual. (G) GO term analysis of marker genes for the MT2^high^ spot. (H) Representative spot distribution of spatial transcriptomics data from a young representative monkey ovary. Left, the spatial distribution of spots within follicle and non-follicle regions. Right, the spatial distribution of each spot type. (I) Representative spatial distribution of different spot types in ovarian tissue of a young monkey. Each spot type is separately represented.

In this study, we performed spatial transcriptomics sequencing (Visium, 10× Genomics) of ovaries harvested from four young (4–5-year-old) and four aged (16–18-year-old) NHPs (Fig. 1A). To this end, we first performed H&E staining and imaging observation of O.C.T compound-embedded sections from the whole intact ovary tissue, containing the cortex and medulla, followed by penetration, RNA isolation, and spatial localization barcode sequencing. For the 10× Genomics Visium technology used in the study, which offers resolution of approximately 55 μm per spot, each section contains up to 4,992 spots in the capture area (6.5 mm by 6.5 mm). After quality control, we obtained high-quality spatial transcriptomic data capturing 17,393 spots (with the median sequencing depth of a single spot at around 200,000 reads and 2,000 genes), and loaded each sample into the Giotto package for normalization (Fig. S1A–E). Upon unsupervised clustering of spots from all samples, we identified nine transcriptionally distinct groups, and we used both the uniform manifold approximation and projection (UMAP) and the physical space of the tissue to present the spatial distribution of those spots (Fig. 1B–I). Based on several canonical marker genes and scoring diverse collections of marker genes for ovarian cell types, combined with the structural characteristics of ovarian tissue, we identified the major cells types locating in the spots and classified them into two regions: (i) follicle region, including oocyte (OO, marked by *DDX4* and *GDF9*, 3.8%), granulosa cell (GC, marked by *INHBB* and *AMH*, 9.6%), theca cell (TC, marked by *STAR* and *CYP17A*, 13.0%), and primordial and primary follicles mixed spots (PPF, marked by the markers of oocyte and GC, 7.8%); (ii) non-follicle region, including ovarian surface epithelium (OSE, marked by *ALDH1A1* and *KRT19*, 2.9%), stromal cell (SC, marked by *TCF21* and *ARID5B*, 41.2%), smooth muscle cell (SMC, marked by *TAGLN* and *ACTG2*, 8.53%), and immune cell (IM, marked by *CD68* and *LYZ*, 7.75%) (Figs. 1C, 1D, and S2). Functional annotation enrichment analysis of the cell type-specific marker genes revealed the consistency of transcriptional characteristics and physiological functions (Fig. 1E). In addition, we identified an unappreciated spot in which *MT2* (Metallothionein 2) and *TIMP1* (*TIMP* metallopeptidase inhibitor 1) were highly expressed. Specifically, the gene expression in this spot was functionally linked to response to hormones (*AREG*, *FOS*, *INHBA*), vasculature development (*ACTG1*, *ENG*, *VEGFC*), inflammatory response (*SERPINA3*, *C3*, *S100A9*), and extracellular matrix organization (*COL1A1*, *LUM*, *FN1*) (Fig. 1F and 1G), and we thus named it MT2^high^ spot (MT2^high^, 5.5%). More importantly, all cell types were distributed at the corresponding spatial locations, indicating that the spatial transcriptomic data delineated the spatial structure of the ovaries well. For example, OSE was predominantly localized in the periphery of the ovarian cortex (Fig. 1H and 1I), whereas within the ovarian follicle, OO was surrounded by GC, which in turn was adjacent to TC in secondary and antral follicles (Fig. 1H and 1I). In addition, SMC and IM were interspersed throughout the whole ovarian tissue, with some of them located around the blood vessels (Fig. 1H and 1I). Collectively, our spatiotemporal transcriptomic atlas decodes the spatially resolved cell types of the NHP ovary.

### Age-related spatial architecture disorganization in NHP ovary

**Figure 2.**
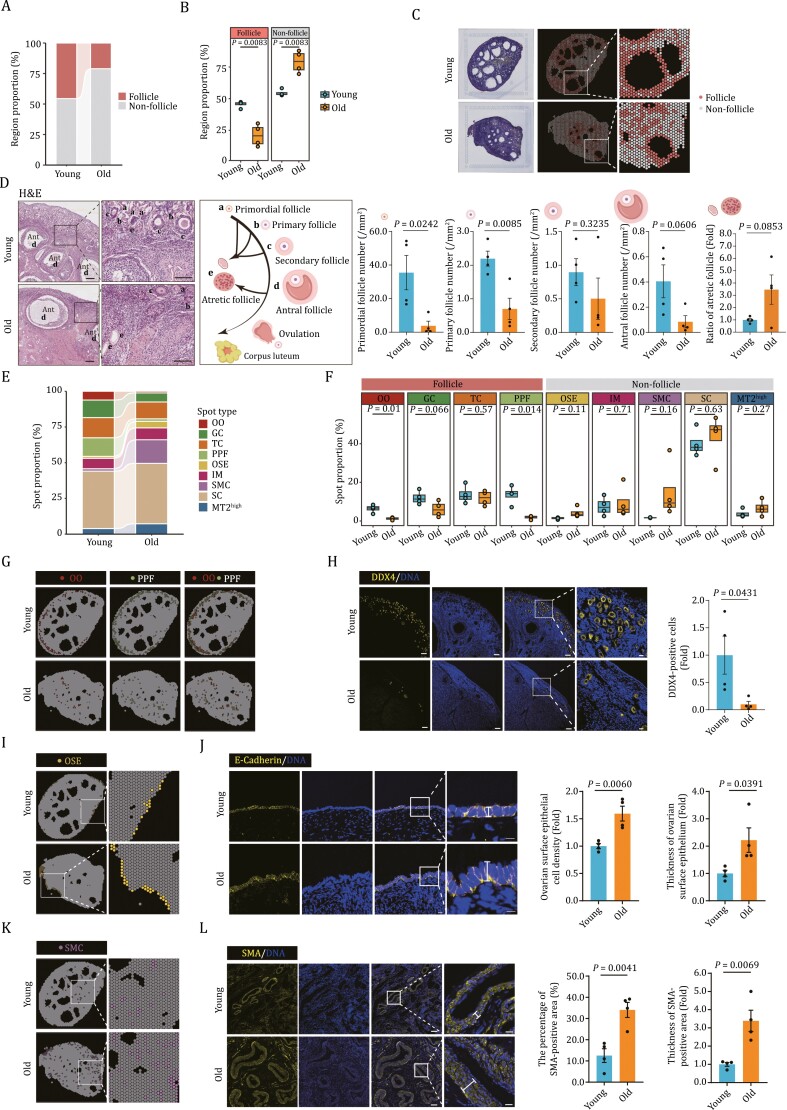
Age-related structural disorganization in NHP ovary. (A) Bar plot showing the proportion distribution of follicle and non-follicle regions of young and old monkey ovaries. (B) Box plots showing the proportion of indicated regions in young and old monkey ovaries. (C) The distribution of follicle and non-follicle regions assigned onto the sections of young and old monkey ovaries. (D) Representative H&E staining images showing the follicles at different developmental stages in young and old monkey ovaries. The antral follicle is abbreviated as “Ant”. a, b, c, d, and e denote primordial, primary, secondary, antral, and atresia follicles, respectively. Number of primordial, primary, secondary, antral follicles per square millimeter and the percentage of atresia follicles are shown on the right. Scale bars, 200 μm and 100 μm (zoomed-in images). *n* = 4 monkeys for each group. (E) Bar plot showing the spot type proportions in young and old monkey ovaries. (F) Box plots showing the proportion of each spot type in young and old ovaries. (G) UMAP plot showing the distribution of OO and PPF spots spatially assigned onto the sections of young and old monkey ovaries. (H) DDX4 immunofluorescence staining of ovaries from young and old monkeys. Left, the representative images. Scale bars, 10 μm and 20 μm (zoomed-in images). Right, the numbers of DDX4-positive cells were quantified as fold changes (old vs. young), and presented as mean ± SEMs. *n* = 4 monkeys for each group. (I) The distribution of OSE spot spatially assigned onto the sections of young and old monkey ovaries. (J) E-Cadherin immunofluorescence staining of young and old monkey ovaries. Density of ovarian surface epithelial cells and thickness of OSE were quantified as fold changes (old vs. young) and presented on the right as mean ± SEMs. Scale bars, 10 μm. *n* = 4 monkeys for each group. (K) The distribution of SMC spot spatially assigned onto sections of young and old monkey ovaries. (L) SMA immunofluorescence staining of young and old monkey ovaries. The percentage of SMA-positive area and the thickness of SMA-positive area were quantified (old vs. young) and presented as mean ± SEMs on the right. Scale bars, 100 μm and 20 μm (zoomed-in images). *n* = 4 monkeys for each group.

Next, we sought to analyze the spatial characteristics associated with ovarian aging. Globally, we observed an atrophy of the follicle region and an expansion of the non-follicle region in aged ovaries relative to their young counterparts (Fig. 2A–C). These changes were reflected by prominently reduced spot numbers within the follicular region and increased spot numbers within the non-follicle region (Fig. 2A–C). Consistently, H&E demonstrated an extensive loss of follicles at each developmental stage (primordial, primary, secondary, and antral follicles) in follicle region (Fig. 2D). Further, when we statistically compared the proportion of annotated spot types and their spatial distribution between young and aged individuals, we found in the aged ovaries a decrease in the number of spots associated with OO and PPF, validated by DDX4 (a well-known oocyte marker, also named as VASA) immunostaining (Castrillon et al., 2000), as well as a reduction of follicle-supporting somatic cells, such as GC and TC, which was in concert with previously observed results (Fig. 2E–H). Together with the increased numbers of atretic follicles observed in aged ovaries (Fig. 2D), these findings portray an aging-related decline in ovarian follicle reserves. Moreover, the numbers of other spot types filling the non-follicle region in aged tissues, including OSE, SC, and SMC, trended towards an increase (Fig. 2F). To experimentally validate our findings, we conducted E-cadherin immunofluorescence analysis, a marker specific to the OSE (Auersperg et al., 2001; Zhang et al., 2020). Our results revealed an increased thickness of the E-cadherin-positive region in aged ovaries (Fig. 2I and 2J), which supports a contribution to elevated tissue stiffness associated with ovarian aging (Amargant et al., 2020). Additionally, the increased numbers of SMA-positive SMCs and the decreased numbers of CD31-positive endothelial cells in aged ovaries support that the tunica media thickens whereas the tunica intima within blood vessels becomes compromised (Figs. 2K, 2I, and S3), phenotypes consistent with blood vessel aging and dampening the nutrition supply (Brown and Russell, 2014). Altogether, these data portray a comprehensive spatiotemporal landscape of the tissue disorganization occurring during NHP ovarian aging.

### The global view of spatially resolved transcriptomic profiles of non-human primate ovarian aging

**Figure 3.**
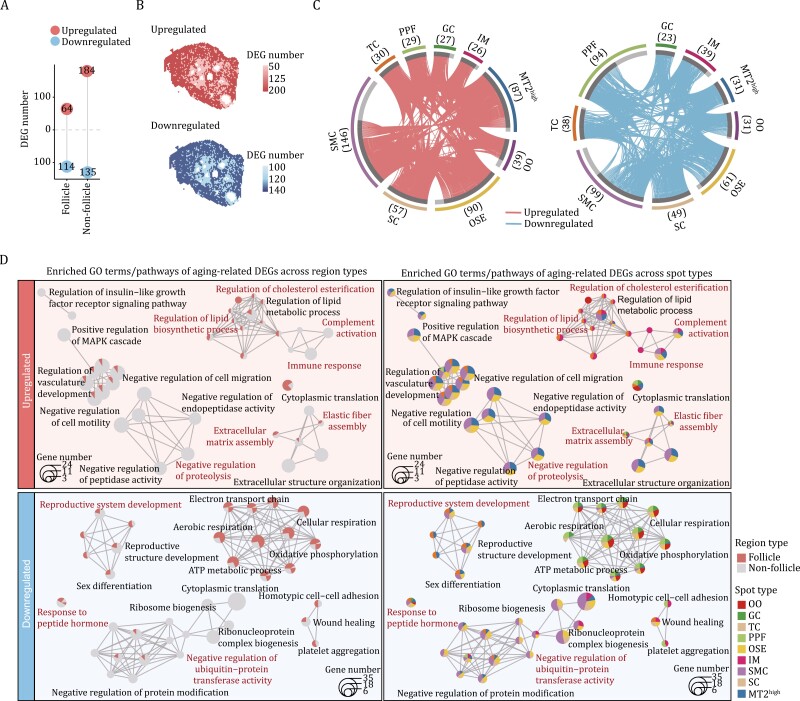
Age-related changes in spatial transcriptomic profiling of NHP ovary. (A) Lollipop plot showing the number of aging-related differentially expressed genes (aging DEGs, old vs. young) in the follicle and the non-follicle regions. (B) Spatial distribution of the number of up- and down-regulated aging DEGs in the follicle and the non-follicle regions. (C) Circos plots showing the aging DEGs for each spot type. Each connecting curve represents one common aging DEG shared in two spot types. The number of aging DEGs for each spot type was also annotated in the corresponding parentheses. (D) Networks visualizing representative GO terms and pathways of aging DEGs across different spot types of monkey ovary. The nodes represent GO terms. The pie charts show the proportion of the number of genes that hit a certain term across spot types.

To spatially resolve the transcriptomic profiles of non-human primate ovarian aging, we first identified the age-associated differentially expressed genes (DEGs) (aging DEGs for abbreviation) (|LogFC| > 0.5 and FDR < 0.05) across different spots between old (O) and young (Y) individuals. The results showed that SMC, OSE, and MT2^high^ rank as the top three spot types harboring the higher numbers of upregulated DEGs (146, 90, and 87 genes, respectively), while SMC, PPF, and OSE spots rank as top three with higher number of downregulated DEGs (99, 94, and 61 genes, respectively) (Fig. 3A–C). These findings underscore that somatic cells are more susceptible to aging, likely forming a dysregulated microenvironment following on from compromised follicle development and contributing to ovarian aging.

Through a functional annotation enrichment analysis of aging-induced DEGs across two major regions and all spots, we aimed to determine the molecular basis most affected by aging. In alignment with the aforementioned observations, we discovered a profound enrichment of altered pathways in ovarian somatic cells within non-follicle region compared to those in follicle region (Fig. 3D). Notably, several characteristics recognized as classical hallmarks of aging (López-Otín et al., 2023), were detected in most spots belonging to the non-follicle region, including (i) aggravated inflammatory response pathways (i.e., immune response and complement activation), (ii) increased extracellular matrix accumulation (i.e., elastic fiber assembly and extracellular matrix assembly), (iii) dysregulation of lipid metabolism (i.e., upregulated genes converged on regulation of lipid biosynthetic process and regulation of cholesterol esterification), (iv) loss of proteostasis (i.e., upregulated genes involving in negative regulation of proteolysis, and downregulated genes correlated to negative regulation of ubiquitin-protein transferase activity) (Fig. 3D).

Although lower number of aging DEGs in the spots within follicle region and particularly for OO may suggest a relatively stable transcriptional state affected by aging in germ cell, we still discovered molecular features predominantly in spots within the follicle region (Fig. 3D), such as downregulated DEGs associated with the electron transport chain (Fig. 3D), which may compromise energy supply and underlie age-related follicle degeneration. Strikingly, several pathways specific for the ovarian functions were broadly altered in spots across the ovary, that is, including both follicle and non-follicle regions. For instance, downregulated DEGs included genes broadly correlated to reproductive function (i.e., reproductive system development and response to peptide hormone) (Fig. 3D), as well as wound healing, a crucial mechanism for ovarian tissue repair after ovulation (Mara et al., 2020), consistent with declined ovarian function in aging.

Taken together, the findings revealed that ovarian somatic cells likely cause a hostile microenvironment detrimental to ovarian function, and how directed cellular and molecular clues target ovarian aging at spatial resolution.

### Spatial transcriptomics unveils the senescence signatures in NHP ovarian aging

**Figure 4.**
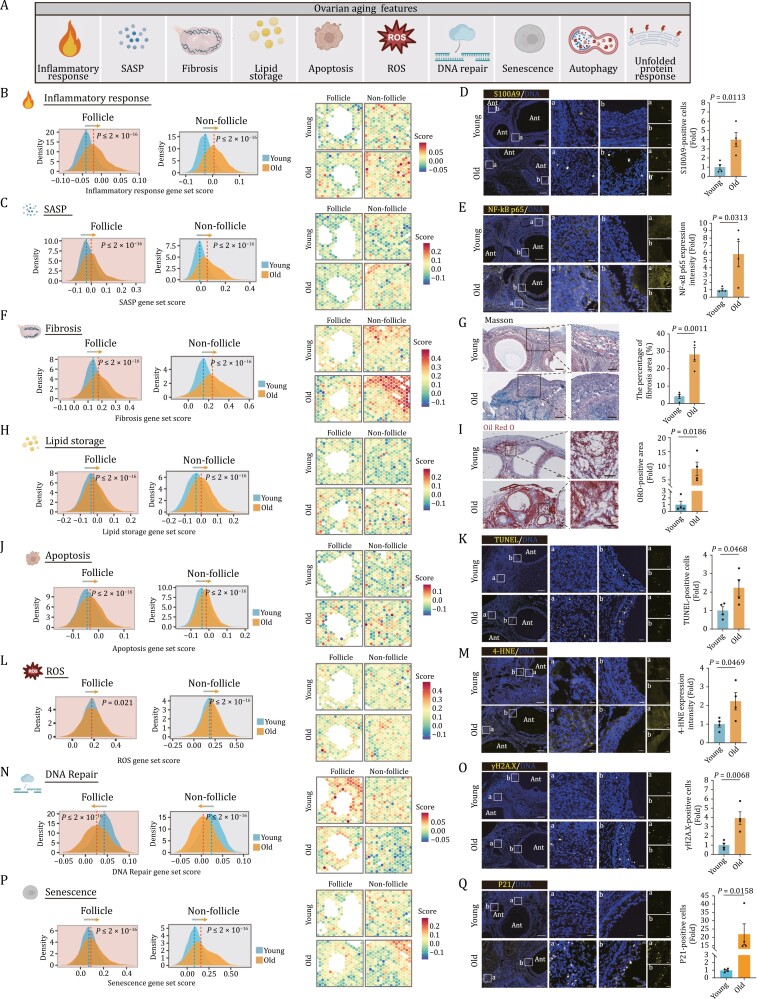
Transcriptional changes of several canonical aging hallmarks in NHP ovarian aging. (A) Schematic diagram showing the ovarian aging features unveiled by spatial transcriptomics. (B) Ridge plots (left) and spatial visualization (right) showing the global distribution density of gene set score of inflammatory response-related genes in young and old groups. The corresponding dashed lines represent the median of each group. (C) Ridge plots (left) and spatial visualization (right) showing the global distribution density of gene set score of SASP-related genes in young and old groups. The corresponding dashed lines represent the median of each group. (D) S100A9 immunofluorescence staining of ovaries from young and old monkeys. Representative images are shown on the left. Among them, “a” represents the staining in the non-follicle region, and “b” represents the staining in the follicle region. Scale bars, 200 μm and 20 μm (zoomed-in images). The numbers of S100A9-positive cells were quantified as fold changes (old vs. young), and presented as mean ± SEMs on the right. *n* = 4 monkeys for each group. (E) NF-κB p65 immunofluorescence staining of ovaries from young and old monkeys. Representative images are shown on the left. Among them, “a” represents the staining in the non-follicle region, and “b” represents the staining in the follicle region. Scale bars, 200 μm and 20 μm (zoomed-in images). The relative intensity was quantified as fold changes (old vs. young), and presented as mean ± SEMs on the right. *n* = 4 monkeys for each group. (F) Ridge plots (left) and spatial visualization (right) showing the global distribution density of gene set score of fibrosis-related genes in young and old groups. The corresponding dashed lines represent the median of each group. (G) Masson’s trichrome staining of young and old monkey ovaries. Representative images are shown on the left. Scale bar, 200 μm and 100 μm. The percentage of fibrosis area was quantified (old vs. young), and presented as mean ± SEMs on the right. *n* = 4 monkeys for each group. (H) Ridge plots (left) and spatial visualization (right) showing the global distribution density of gene set score of lipid storage-related genes in young and old groups. The corresponding dashed lines represent the median of each group. (I) ORO staining of ovaries from young and old monkeys. Representative images are shown on the left. Scale bars, 200 μm and 100 μm (zoomed-in images). The percentage of ORO-positive area was quantified was quantified as fold changes (old vs. young), and presented as mean ± SEMs on the right. *n* = 4 monkeys for each group. (J) Ridge plots (left) and spatial visualization (right) showing the global distribution density of gene set score of apoptosis related-genes in young and old groups. The corresponding dashed lines represent the median of each group. (K) TUNEL staining of ovarian tissues from young and old monkeys. Representative images are shown on the left. Among them, “a” represents the staining in the non-follicle region, and “b” represents the staining in the follicle region. Scale bar, 200 μm and 20 μm (zoomed-in images). The numbers of TUNEL-positive cells in the tissues were quantified as fold changes in old ovaries versus in young counterparts, and shown as mean ± SEMs on the right. *n* = 4 monkeys for each group. (L) Ridge plots (left) and spatial visualization (right) showing the global distribution density of gene set score of ROS-related genes in young and old groups. The corresponding dashed lines represent the median of each group. (M) 4-HNE staining of ovary tissues from young and old monkeys. Representative images are shown on the left. Among them, “a” represents the staining in the non-follicle region, and “b” represents the staining in the follicle region. The immunofluorescence expression intensity in the tissues was quantified as fold changes (old vs. young), and shown as mean ± SEMs on the right. *n* = 4 monkeys for each group. Scale bar, 200 μm and 20 μm (zoomed-in images). (N) Ridge plots (left) and spatial visualization (right) showing the global distribution density of gene set score of DNA repair-related genes in young and old groups. The corresponding dashed lines represent the median of each group. (O) γH2A.X staining of ovary tissues from young and old monkeys. Representative images are shown on the left. Among them, “a” represents the staining in the non-follicle region, and “b” represents the staining in the follicle region. γH2A.X-positive cells in the tissues were quantified as fold changes (old vs. young), and shown as mean ± SEMs on the right. *n* = 4 monkeys for each group. Scale bar, 200 μm and 20 μm (zoomed-in images). (P) Ridge plots (left) and spatial visualization (right) showing the global distribution density of gene set score of senescence-related genes in young and old groups. The corresponding dashed lines represent the median of each group. (Q) P21 staining of ovary tissues from young and old monkeys. Representative images are shown on the left. Among them, “a” represents the staining in the non-follicle region, and “b” represents the staining in the follicle region. P21-positive cells in the tissues were quantified as fold changes (old vs. young), and shown as mean ± SEMs on the right. *n* = 4 monkeys for each group. Scale bar, 200 μm and 20 μm (zoomed-in images).

Based on the profiling described above, we next performed an in-depth analysis of the expression changes of gene sets corresponding to a range of classic aging hallmarks (Aging Biomarker et al., 2023) in aged NHP ovaries (Fig. 4A). Given the aforementioned observations, we first calculated gene scores associated with inflammatory responses and the senescence-associated secretory phenotype (SASP), both well known to elicit chronic inflammation and facilitate aging (Birch and Gil, 2020; Sun et al., 2022), and examined their spatial distribution in young and aged NHP ovaries. We found an age-dependent upregulation of genes in these two gene sets across ovarian spots within both follicle and non-follicle regions (Fig. 4B and 4C). Consistently, in aged ovaries, we found a massive infiltration of CD163^+^ macrophages, as well as an accumulation of NF-κB p65^+^ cells and cells positive for S100A9, an inflammatory factor that is recently considered to be involved in cellular senescence (Ma et al., 2020; Zhang et al., 2023) (Figs. 4D, 4E, and S4A–C). Similarly, we observed upregulation of genes closely linked to fibrosis and the TGF-β-signaling pathway in the aged spots relative to those in young groups, and validated the presence of elevated collagen fibers-enriched fibrosis areas, as indicated by Masson’s Trichrome staining (Figs. 4F, 4G, S4D, and S4E), supporting age-associated accumulation of extracellular matrix. In addition, we noticed a higher score for a gene set associated with lipid storage in the aged groups compared to that in young counterparts, consistent with data showing augmented deposition of lipid by Oil Red O (ORO) staining (Figs. 4H, 4I, and S4F). Furthermore, we examined the transcriptional perturbance of three branching pathways of the UPR, namely ATF6, IRE1, and PERK signaling (Read and Schröder, 2021), as well as autophagy pathways known to degrade unfolded or misfolded proteins (Cheng and Liu, 2019) (Fig. S4G–J). These results indicate that both the PERK signaling and autophagy pathways are activated with advanced age (Fig. S4I and J). To be noted, apoptotic pathway, the major downstream cascade of PERK signaling was also activated with age (Figs. 4J and S4K), suggesting that the cells failed to restore the balance between persistent aberrant misfolded protein refolding and unfolded protein clearance, and eventually destined for cell death. In line with these analyses, the proportion of TUNEL-positive and cleaved-caspase-3-positive apoptotic cells markedly increased in aged ovaries relative to those in young tissues (Figs. 4K and S4L). Moreover, the expression levels of genes responsible to produce reactive oxygen species (ROS) were elevated, which we experimentally validated by detecting an accumulation of lipid peroxidation lesion marked by 4-HNE in aged ovaries (Figs. 4L, 4M, and S5A). As genomic instability often instigates DNA damage, we further analyzed gene expression alterations related to DNA repair and found dampened DNA repair capability associated with advanced age (Figs. 4N and S5B). Accordingly, we observed increased γH2A.X-positive cells in aged ovaries (Fig. 4O). Finally, we found that aged ovarian cells acquired senescence signatures in response to aging and consistently noticed increased SA-β-Gal-positivity and P21-positive senescent cells in aged ovaries (Figs. 4P, 4Q, S5C, and S5D). Overall, these findings showcased a broad aging-associated transcriptional program, coinciding with a cascade of tissue structural deterioration in aged NHP ovaries.

### Identification of aging hotspot in NHP ovary from spatial aspect

**Figure 5.**
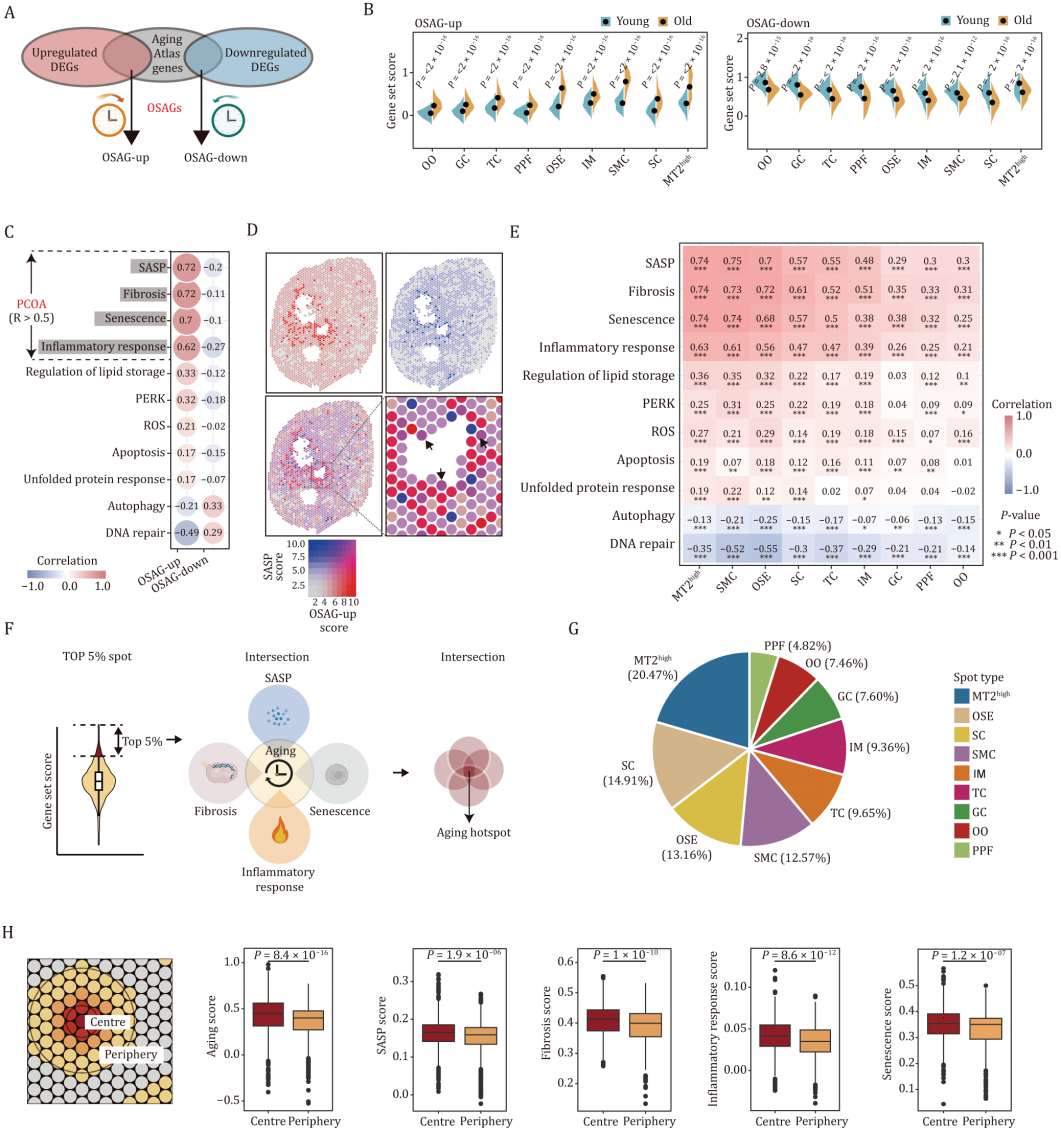
Identification of aging hotspot in NHP ovary from spatial aspect. (A) Schematic diagram showing the generation of OSAG gene sets. (B) Violin plots showing the gene set scores of OSAG-upregulated and OSAG-downregulated genes across different spot types from old and young monkey ovaries. (C) Pearson correlation analysis between OSAG-upregulated or OSAG-downregulated gene set scores and gene set scores of 11 age-related signaling pathways in monkey ovary. (D) The spatial distribution and colocalization visualization of spots with OSAG-upregulated gene set score or SASP-related gene set score. (E) Correlation analysis between OSAG-upregulated set scores and gene set scores of 11 age-related signaling pathways across different spot types in monkey ovary. (F) Schematic diagram showing the procedure for identifying aging hotspots based on primary contributors to ovarian aging (PCOA) scores. (G) The proportion analysis of different spot types in total identified aging hotspots. (H) Schematic diagram showing the center and periphery regions around aging spot (left). The bar plots showing the negative correlation between spatial distance from the hotspot and aging or PCOA scores (right).

Next, we ask if there is aging hotspot within ovarian tissue that instigates NHP ovarian aging. To this end, we performed an integrated analysis of aging DEGs across all ovarian spots and genes from the Aging Atlas database (Aging Atlas, 2021) to generate a set of ovary-specific aging genes (OSAGs) (Fig. 5A). Upregulated OSAGs may accelerate the ovarian aging while downregulated OSAGs may delay ovarian aging. As expected, we noticed a series of remarkable changes across all spots in aged groups when we scored the upregulated and downregulated gene sets, respectively (Fig. 5B). We next calculated the correlation index of gene set scores between OSAGs and other gene sets that may distinctly contribute to impairments of ovarian functions. In fact, we did notice positive correlation of scores between upregulated OSAGs and a panel of other gene sets including SASP, fibrosis, senescence, inflammatory response, and so on (Figs. 5C and S6A), as well as positive correlation of scores between downregulated OSAGs and genes involving in autophagy and DNA repair (Fig. 5C). Among them, we observed a higher relevance (over 0.5) between scores of OSAGs and SASP, fibrosis, senescence, and inflammatory response scores (Fig. 5C), suggesting that those pathways serve as the primary contributors to ovarian aging (PCOA), and we thus named these PCOA. Indeed, when considering the spatial distribution aspect, we observed a marked colocalization between spots with a higher upregulated OSAGs score and those with a higher SASP score (Fig. 5D), and did so for other PCOA scores (Fig. S6B). Of particular importance, when we attributed those scores to all the ovarian spots, we found that MT2^high^, SMC, and OSE ranked as the top three spots with a higher correlation to those PCOA in terms of transcriptional profiles (Figs. 5E and S6C), implying their higher susceptibility to aging.

Based on the observations, we next tried to determine the aging hotspot in the NHP ovary at a spatial level. To achieve this, we first calculated the spatial aging score by integrating the upregulated and downregulated OSAGs. Next, through a conjoint analysis of the spots colocalized with the highest aging score and other PCOA scores (ranking top 5% for each), we defined the aging hotspot in the NHP ovary, which is characterized by high levels of SASP, fibrosis, senescence, and inflammatory response (Fig. 5F). We next asked what identity of the aging hotspot is. Through calculating the proportion of each spot type in the total 532 colocalized spots that had acquired the PCOA transcriptional traits, we found that the MT2^high^ occupied around 20.47% colocalized spots (Fig. 5G), along with SC and OSE occupied 14.91% and 13.16%, respectively, implying MT2^high^ may serve as the major senescent foci across the ovarian tissue. As MT2^high^ is featured by high level of inflammation and SASP (Fig. 5E), these findings further implied that MT2^high^ may constitute the primary aging hotspot that disseminates and spreads the aging signaling throughout the whole ovary tissue. In fact, we noticed a marked negative correlation of ovary-specific aging scores with distances from the hotspot, that is, the aging scores gradually declined from the hotspot to the nearby region (Fig. 5H), which was also seen for SASP score (Fig. 5H).

### Characterization of the aging hotspot and its microenvironment in aged ovaries

**Figure 6.**
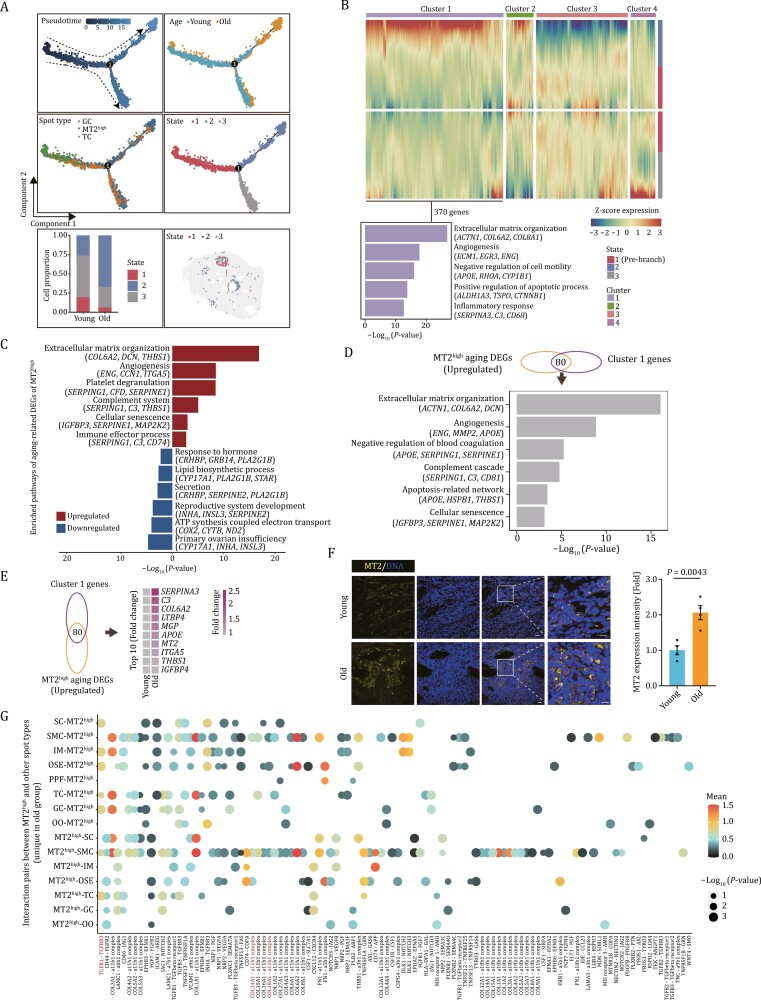
Characterization of the senescence hotspot and its microenvironment in aged ovaries. (A) Pseudotime analysis of GC, TC, and MT2^high^ spot in NHP ovary. Upper left, pseudotime scores of the three spot types in monkey ovary. Upper right, the distribution of young and old groups along the pseudotime trajectory. Middle left, the distribution of these three spot types along the pseudotime trajectory. Middle right, the distribution of different states of MT^high^ spot along the pseudotime trajectory. Lower left, bar plot showing the proportion distribution of three states of MT2^high^ spots between young and old groups. Lower right, the spatial distribution of three states of MT2^high^ spots. (B) Heatmap showing four different expression patterns along the pseudotime trajectory based on branched expression analysis modeling (BEAM) analysis and bar plot showing the enriched GO terms for cluster 1 genes. (C) Enriched GO terms and pathways of aging DEGs in MT2^high^ spot. (D) Bar plot showing pathway enrichment analysis of overlapped genes between upregulated aging DEGs in MT2^high^ spot and Cluster1 genes. (E) Heatmap showing the expression levels of the top 10 (LogFC) overlapping genes between upregulated aging DEGs in MT2^high^ spot and Cluster1 genes. (F) MT2 immunostaining in young and old monkey ovaries. The relative intensity was quantified as fold changes (old vs. young), and presented as mean ± SEMs on the right. Scale bars, 20 μm and 10 μm (zoomed-in images). *n* = 4 monkeys for each group. (G) Dot plot showing the interaction pairs between MT2^high^ and other spot types unique in the aged group.

Interestingly, when we thoroughly examined the spatial locus of MT2^high^ spots, we found that some of them localized in the region nearby the follicular antrum, while some were dispersedly distributed in the stromal region of ovary (Fig. 1H and 1I). To track the MT2^high^ state and explore how it impacts ovarian aging, we performed pseudotime analysis using a combination of the major somatic cells involved in folliculogenesis (Fig. 6A). The results showed that the majority of MT2^high^ located in the later stage along the trajectory following GC and TC (Fig. 6A), implying that MT2^high^ may contain cells differentiation from GC or TC. Intriguingly, MT2^high^ was found to split into three states, one of which accumulated in the aged group (state 2) compared with the other two states (Fig. 6A). By employing trajectory-based differential expression analysis, we identified 370 upregulated genes facilitating the state 2 MT2^high^ (gene cluster 1) (Fig. 6B). Functional annotation of genes in cluster 1 elucidated elevated expression of genes associated with extracellular matrix organization (*ACTN1*, *COL6A2*, *COL8A1*), angiogenesis (*ECM1*, *EGR3*, *ENG*), positive regulation of apoptotic process (*ALDH1A3*, *TSPO*, *CTNNB1*), and inflammatory response (*SERPINA3*, *C3*, *CD68*) (Fig. 6B).

Further, we analyzed age-associated gene expression changes in the MT2^high^ spot and found that upregulated DEGs were closely related to extracellular matrix organization (*COL6A2*, *DCN*, *THBS1*), angiogenesis (*ENG*, *CCN1*, *ITGA5*), immune effector process (*SERPING1*, *C3*, *CD74*), and cellular senescence (*IGFBP3*, *SERPINE1*, *MAP2K2*), while downregulated DEGs were functionally linked to reproductive system development (*INHA*, *INSL3*, *SERPINE2*) and response to hormone *(CRHBP*, *GRB14*, *PLA2G1B*) (Fig. 6C). When we performed an integrative analysis of age-associated upregulated DEGs in the MT2^high^ spot and gene cluster 1, we found a massive overlap between them, manifested by an abundance in pathways related to extracellular matrix organization, apoptosis, angiogenesis, inflammation, and cellular senescence (Fig. 6D). The top 10 overlapped genes included *SERPINA3*, *C3*, *COL6A2*, *LTBP4*, *MGP*, *APOE*, *MT2*, *ITGA5*, *THBS1*, and *IGFBP4*, most of which are associated with inflammatory response and fibrosis (Bai et al., 2022; Isola et al., 2023; Lin et al., 2022) (Fig. 6E). Indeed, we validated the upregulated expression level of MT2 by immunostaining in aged ovaries (Fig. 6F). Overall, these findings suggest that an accumulation of age-dependent MT2^high^ spot particularly under state 2, with an extensive inflammatory level in aged ovaries, may drive the formation of an ovarian aging hotspot to facilitate an inflamed and hostile ovarian niche, diminishing ovarian physiological function and prompting ovarian aging.

To dissect age-related changes in the hotspot and its microenvironments in NHP ovary, we performed cell–cell interaction analysis between MT2^high^ and surrounding spots and discovered pronounced dysregulated cell–cell interactions between MT2^high^ and other spot types (Fig. 6G). Specifically, increased ligand-receptor pairs between aged MT2^high^ and other ovarian spots were highly relevant to fibrosis (Fig. 6G). The representative pairs were COL14A1-a11b1, COL18A1-a11b1, and TGFB1-TGFBR3 (Fig. 6G), likely to cause the instability of the supportive microenvironment, and facilitate the formation of corpus albicans (Sasano and Suzuki, 1997), ultimately leading to ovarian aging. These data implied that MT2^high^ serves as one of the major contributors to hostile microenvironment in aged ovaries.

### APOE serves as an aging hallmark of the primate ovary revealed by spatiotemporal transcriptomics

**Figure 7.**
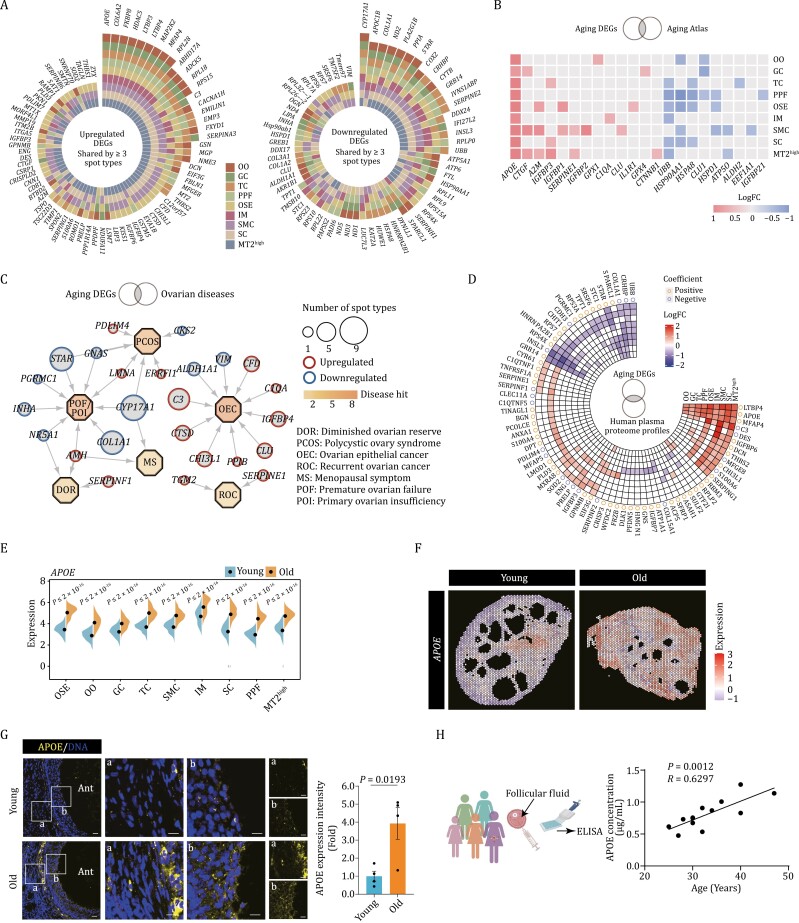
Signatures of NHP ovarian aging based on spatial transcriptome. (A) Plots showing the upregulated and downregulated aging DEGs shared by at least three spot types. (B) Heatmap showing the upregulated and downregulated aging DEGs overlapped with genes annotated in Aging Atlas database across different spot types. (C) Network visualization of upregulated and downregulated DEGs in 11 different ovarian spot types in the ovarian disease. Octagonal nodes represent diseases. Round nodes represent genes and node size positively correlates with the number of spot types differentially expressing the gene, respectively. (D) Circular heatmap showing the upregulated and downregulated aging DEGs overlapped with proteins from human plasma proteome profiles across the lifespan. Correlation coefficient indicates the positive or negative relationship between the indicated proteins with age. (E) Violin plot showing the expression levels of *APOE* in young and old cynomolgus monkey ovaries. (F) The spatial gene expression of *APOE* spatially assigned onto the sections of young and old cynomolgus monkey ovaries. (G) APOE immunofluorescence staining of young and old monkey ovaries. Representative images are shown on the left. Among them, “a” represents the staining in the non-follicle region, and “b” represents the staining in the follicle region. The relative immunofluorescence intensity was quantified as fold changes (old vs. young), and presented as mean ± SEMs on the right. Scale bars, 20 μm and 10 μm (zoomed-in images). *n* = 4 monkeys for each group. (H) ELISA analysis showing the APOE concentration in the follicle fluid of women aged from 25 to 47 years old (*n* = 13).

To further delineate the age-dependent gene expression changes across all spot types, we identified a total of 88 DEGs that were consistently upregulated in at least three spot types, along with 61 DEGs consistently downregulated in at least three spot types (Fig. 7A). Among them, we found that the expression of *APOE*, *COL6A2*, *FKBP8*, *HDAC5*, *LTBP3*, *LTBP4*, *MAP2K2*, *MFAP4*, and *RPL28* were increased, while *CYP17A1*, *APOC1B*, and *COL1A1* were decreased across all the spot types (Fig. 7A). Additionally, we performed an integrated analysis that combined aging DEGs with genes obtained from the Aging Atlas database in order to detect changes in gene expression patterns linked to the aging. Most importantly, we also found that *APOE* exhibited the most dominant upregulation across all the spot types (Fig. 7B).

Next, we then asked whether these aging DEGs are linked to reproductive diseases in ovaries. To this end, we conducted a joint comparative analysis of aging DEGs and genes responsible for a range of ovarian diseases (PCOS, polycystic ovary syndrome; OEC, ovarian epithelial cancer; MS, menopausal symptom; DOR, diminished ovarian reserve; ROC, recurrent ovarian cancer; POF, premature ovarian failure; POI, primary ovarian insufficiency) (Ata et al., 2019; Pal and Santoro, 2002; Tew, 2016) (Fig. 7C). Interestingly, we noticed a prominent enrichment of the overlapped upregulated genes related to inflammatory responses, including serpin serine protease inhibitors (*SERPINE1* and *SERPINF1*) and components of the complement system (*C3* and *C1QA*) (Fig. 7C), suggesting a putative common role of elevation of inflammation in the regulation of NHP ovarian aging and female reproductive diseases. Conversely, several downregulated genes crucial for folliculogenesis were also found to be linked to ovarian diseases, namely, genes for sex hormone biosynthesis such as *STAR*, *CYP17A1*, and *ALDH1A1* (Burris-Hiday and Scott, 2021; Petrosino et al., 2014) (Fig. 7C), along with genes required for follicle development, such as *INHA* (Lovell et al., 2003) (Fig. 7C).

Further, by jointly analyzing the upregulated protein profiles associated with aging in NHP ovarian tissues and human sera (Lehallier et al., 2019), we found a panel of secretory factors APOE, CHI3L1, SERPING1, etc. (Fig. 7D), indicative of their potential application as biomarkers of the degree of ovarian aging. Among them, Apolipoprotein E (APOE) is a protein, vital to lipid metabolism and is involved in pathophysiology of several degenerative disorders such as neurodegenerative diseases (Husain et al., 2021; Zhao et al., 2022). Indeed, *APOE* transcripts were upregulated, along with an increase in protein levels of APOE in aged ovaries (Fig. 7E and 7F). Of note, we also found a colocalization between spots highly expressing *APOE* and those with a higher PCOA score at spatial level (Fig. S7A). Together with a positive correlation of the expression level of *APOE* and *MT2* indicated by Pearson correlation analysis (Fig. S7B), as well as numerous spatially colocalized spots highly expressing *APOE* and *MT2* (Fig. S7C), these findings imply a potential involvement of APOE in ovarian aging.

Intriguingly, given that APOE has recently been recognized as a secreted protein, we next explored whether its level in secreted form in human individuals increases with age (Fig. 7G). As expected, we found that APOE levels were remarkably increased in the follicular fluid of older human individuals compared to younger individuals (Fig. 7H). These findings suggest the potential of APOE as a novel biomarker of human ovarian aging.

## Discussion

Ovarian aging compromises fertility and causes multiple pathological conditions, and unveiling mechanisms of ovarian aging is therefore of both scientific and clinical importance. Pioneering studies of single-cell transcriptomic analyses have dissected the cellular and molecular features of ovarian aging with single-cell accuracy (Ben Yaakov et al., 2023; Cai et al., 2022; Isola et al., 2023; Lengyel et al., 2022; Machlin and Shikanov, 2022; Mishina et al., 2021; Wang et al., 2020; Wei et al., 2023; Yuan et al., 2021). However, biases introduced by digestion steps and the absence of spatial information presents challenges for the use of single-cell omics to determine accurate age-dependent changes corresponding to its spatial locus. The emergence of spatial transcriptome techniques capturing RNA *in situ* can unravel aging-specific gene expression traits with definite cell type and tissue locus information. Moreover, spatial profiling allows the identification of tissue locations most susceptible to aging and their interplay with the microenvironment, which is especially powerful for tissues with a highly heterogeneous architecture such as the ovary (Ma et al., 2023; Shi et al., 2023). Here, by applying spatially resolved transcriptomics, we identify tissue-level transcriptional fingerprints of young and aged primate ovaries, deepening our insight into the cellular and molecular mechanism underlying NHP ovarian aging. While representing a significant advance, it should be noted that spatial transcriptomics still has limited resolution and sequencing depth compared to single-cell transcriptomics. For example, a single spot on 10× Visium Spatial Gene Expression slide is typically 55 μm in diameter and usually contains 5–10 cells, which makes it difficult to annotate the specified cell types and further interpret the cell type-specific transcriptional changes in response to aging. Therefore, further technical improvements are urgently required to define cell types more conclusively and disentangle the cell type-specific regulatory mechanisms underpinning primate ovarian aging.

Intriguingly, we identified MT2^high^ as the major aging hotspot, the primary source of senescence in the NHP ovary, manifesting as the transcriptional traits of inflammation, angiogenesis, and response to hormone. It contains highly specialized ovarian cell types potentially participating in the ovulation, as well as corpus luteum formation, maturation and regression. In support of our results, ovulation is well known to be accompanied by an extensive inflammatory response (Duffy et al., 2019), which is important to activate proteolytic pathways to reorganize the follicular stromal components. Angiogenesis is also critical to shape both luteal structure and function, including to provide the corpus luteum with blood flow supporting its high metabolic rate (Woad and Robinson, 2016). Importantly, we found a state of MT2^high^ spot predominantly in old individuals that acquires apoptosis and cellular senescence signatures. Since ovarian tissue rupture and remodeling periodically occur throughout the ovulation process and luteum phase (Komatsu and Masubuchi, 2017), akin to a physiological injury, we propose that this cycling process may contribute to an age-dependent accumulation of MT2^high^ spot, which not only undergoes apoptosis itself but also promotes ovarian aging by disseminating and amplifying the senescence wave. Although our spatial profiling approach has provided insight into the cellular and molecular landscape of ovarian aging, further investigations are required to deduce the regulatory mechanisms.

Collectively, our study depicts comprehensive transcriptomic signatures of NHP ovarian aging at spatial cell resolution, and identifies an aging hotspot with high-inflammation trait which may escalate the inflammatory cascade and elicit the senescent wave in aging ovaries. Our analysis paves the way for identifying potential diagnostic biomarkers and therapeutic targets against ovarian aging and female reproductive disorders.

## Method details

### Experimental animals

All cynomolgus monkeys (*Macaca fascicularis*) originated from the Beijing Institute of Xieerxin Biology Resource. The experimental animals were kept at around 25°C under conditions of 12 h of light and 12 h of darkness, and were raised at the Beijing Institute of Xieerxin Biology Resource Center, in compliance with all local and federal animal research laws. All animals received a commercial diet twice a day, drank tap water at will, and were fed vegetables and fruits once a day under the careful supervision of a veterinarian. Before the experiment, no animal had an experimental history which might affect physiological aging or increase disease susceptibility. The use of cynomolgus monkeys in this study was approved by the Institutional Animal Care and Use Committee of the Institute of Zoology, Chinese Academy of Sciences, and conducted in accordance with the guidelines for Ethical Treatment of Non-Human Primates. All animal experiments were carried out by certified veterinarians and the whole process complied with the laws governing animal research.

### Tissue collection

Ovarian tissue samples for spatial data analysis were collected from four young (4–5 years old) and four old (16–19 years old) female cynomolgus monkeys. We selected the right ovary and cut it horizontally into two equal-sized halves. One part was fixed with 4% paraformaldehyde (PFA) in preparation for paraffin embedding and histological analysis. The other part was placed in the optimal cutting temperature compound (O.C.T) (Sakura, 4583) and stored in the refrigerator at −80°C. Follicular fluid was obtained from the Tianjin First Central Hospital and Peking University Third Hospital which received assisted reproductive technology programs mainly due to sperm quality issues. Follicular fluid was collected during oocyte retrieval and then centrifuged at 3,000 rpm for 15 min at 4°C to remove precipitation and cells. The supernatant was then transferred to a freezing tube, and immediately snap-frozen in liquid nitrogen and then stored at −80°C for further analysis.

### Tissue Senescence-associated β-galactosidase (SA-β-Gal) staining

SA-β-Gal staining was performed following the previously published protocols (Debacq-Chainiaux et al., 2009; Ma et al., 2020, 2021; Wang et al., 2018). In brief, the O.C.T-embedded ovarian tissues were cryosectioned at a thickness of 8 μm with a Leica CM3050S cryomicrotome, mounted on Superfrost Plus microslides (VWR) and stored at −80°C. Before SA-β-Gal staining, sections were dried at room temperature (RT), fixed in fixation buffer (2% formaldehyde and 0.2% glutaraldehyde) for 5 min and stained with freshly prepared SA-β-Gal staining solution (1 mg/mL X-gal (Amresco, 0428), 5 mmol/L K_4_[Fe(CN)_6_], 5 mmol/L K_3_[Fe(CN)_6_], 2 mmol/L MgCl_2_, 150 mmol/L NaCl, 40 mmol/L citric acid/Na phosphate buffer) at 37°C for 2 weeks. Further, the sections were counterstained with Nuclear Fast Red Staining Solution (Beyotime, C0151) to visualize the nucleus. Finally, the sections were dehydrated and sealed with resinous mounting medium. Images were taken with Olympus CKX41 microscope imaging system, and the SA-β-Gal-positive areas were quantified with ImageJ.

### Immunofluorescence staining

Immunofluorescence staining was conducted as previously described (Li et al., 2021; Ma et al., 2021; Wang et al., 2022). In brief, the ovarian tissue sections embedded in paraffin (5 μm thickness) were initially deparaffinized using xylene and subsequently rehydrated through a series of alcohol dilutions (100%, 100%, 95%, 80%, 75%). After being washed in distilled water, the sections were immersed in citrate buffer for antigen retrieval at 115°C/10 min using a pressure cooker. Following this, they were cooled to RT, and subsequently rinsed in PBS for three times. Sections were permeabilized with 0.4% Triton X-100 for 30 min, followed by three subsequent washes in PBS. Subsequently, sections were incubated with blocking buffer (10% donkey serum in PBS) at RT for 1 h, primary antibodies overnight at 4°C, and fluorescence-labeled secondary antibodies at RT for 1 h. Hoechst 33342 (Thermo Fisher, H3570, 1:1,000) was utilized for nuclear visualization as a counterstain. Finally, the sections were mounted using VECTERSHIELD^®^ anti-fading mounting medium (Neobioscience, H-1000), and image capture was performed using the Zeiss LSM900 confocal system. The antibodies used for immunofluorescence staining in this study are detailed below: p21^Waf1/Cip1^ Rabbit mAb (Cell Signaling Technology, 2947S, 1:200), Anti-Ki67 antibody (Abcam, ab15580, 1:200), Anti-4 Hydroxynonenal antibody (Abcam, ab46545, 1:400), Cleaved-Caspase-3 antibody (Cell Signaling Technology, 9661S, 1:200), Phospho-H2AX-S139 Rabbit mAb (ABclonal, AP0687, 1:200), Anti-Apolipoprotein E antibody (Abcam, ab183597, 1:200), Human/Mouse E-Cadherin Affinity Purified Polyclonal Ab (R&D systems, AF748, 1:400), Anti-alpha smooth muscle Actin antibody (Abcam, ab150301, 1:200), Recombinant Anti-CD163 antibody (Abcam, ab182422, 1:200), NF-kappaB p65 (D14E12) XP® Rabbit mAb (Cell Signaling Technology, 8242S, 1:200), Anti-S100A9 antibody (Abcam, ab92507, 1:200), CD31/PECAM1 Rabbit pAb (ABclonal, A0378, 1:200), Anti-DDX4/MVH antibody (Abcam, ab13840, 1:200), and MT2A Rabbit pAb (ABclonal, A2018, 1:100). Secondary antibodies used were the following: donkey anti mouse-AF488 (Thermo Fisher, A21202, 1:500), donkey anti rabbit-AF488 (Thermo Fisher, A21206, 1:500), and donkey anti goat-AF488 (Invitrogen, A11055, 1:500).

### Masson’s trichrome staining

Masson’s trichrome staining was performed as previously described (Huang et al., 2022). In brief, the ovarian tissue sections embedded in paraffin (5 μm thickness) were initially deparaffinized using xylene and subsequently rehydrated through a series of alcohol dilutions (100%, 100%, 95%, 80%, 75%). Following the rinse in distilled water, the sections were immersed in Mordant Solution at 60°C for 1 h and then rinsed in flowing water for 10 min. Then, the sections were incubated consecutively in Celestite Blue Solution and Mayer Hematoxylin Solution for 3 min. They were then slightly washed with distilled water twice, each time for 30 s. Next, the sections were differentiated by acid differentiation solution for several seconds and rinsed in flowing water for 10 min. Ponceau-Acid Fuchsin Solution was then applied to the sections for a 10-min staining period. After two subsequent washes, the sections were then treated with Phosphomolybdic Acid Solution for 10 min. Following the removal of the excess dye solution, the sections were directly stained with Aniline Blue Solution for 5 min. After differentiation in 1% acetic acid solution for 2 min, the sections were dehydrated and mounted using a resinous mounting medium. Images were carried out with PerkinElmer Vectra Polaris.

### Hematoxylin and eosin staining

H&E staining was performed as previously described (Wang et al., 2020). Briefly, the embedded ovarian tissues were cut into 5 μm sections using a rotary microtome and dried at 60°C for 24 h. Then, the paraffin-embedded ovarian tissue sections were deparaffinized in xylene and rehydrated through a series of alcohols (100%, 100%, 95%, 80%, 75%). Following a brief wash in distilled water, the sections were incubated with hematoxylin solution for 3 min and then washed with running tap water to remove excess hematoxylin. Subsequently, the sections were differentiated in 1% acid alcohol for 5 s and washed with flowing water for 10 min. Following this, the sections were incubated in the eosin counterstain, subsequent were dehydrated quickly in a graded series of ethanol (85%, 95%, 100%, 100%), and immersed in xylene. Finally, the slides were covered with Cytoseal-60 (Stephens Scientific, USA).

### Follicle counting

Follicle counting was performed using the H&E-stained ovary sections as previously described with minor modifications (Wang et al., 2020). The primordial follicles were defined as an oocyte peripherally surrounded by a single flattened layer follicle cells and are generally positioned closer to the epithelium. The primary follicles were an enlarged oocyte surrounded by a single layer of cuboidal follicle cells, and a follicular membrane composed of connective tissue appeared at the periphery of the follicles. Secondary follicles were characterized as harboring an enlarged oocyte surrounded by at least a partial or complete two-layer cuboidal GCs. Antral follicles were characterized by the presence of a single follicular cavity filled with follicular fluid. Follicular atresia occurred at any stage of follicular development. Early follicular atresia was characterized by the oocyte degenerating (convoluted and condensed or fragmented) or absent. When atresia occurred in secondary follicles or antral follicles, oocytes were degenerated and disappeared, GCs were loosely detached, and follicular membrane cells enlarged to form interstitial glands. In H&E staining, it showed a strong eosinophilic red color. We counted primordial follicles, primary follicles, secondary follicles, and antral follicles in three repetitions at intervals of 15 consecutive sections. The follicular density was calculated by the mean number of follicles divided by the area (mm^2^) of ovarian tissue. Then the percentage of atretic follicles to total follicles (including healthy follicles and atretic follicles at each stage) was calculated.

### Enzyme-linked immunosorbent assay

ELISA was performed as instructions described (Abcam, ab108813). Firstly, standard samples were prepared according to the described content for a standard curve. Next, 50 μL of Apolipoprotein E standard or the designated samples were introduced into appropriate wells and subjected to an incubation for 2 h in microplates. Subsequently, a manual washing procedure involving five cycles with 200 μL of 1× wash buffer was carried out. Following this, 50 μL of 1× Biotinylated Apolipoprotein E antibody was added to each well and subjected an incubation for 1 h, followed by the aforementioned microplate washing process. Then, 50 μL of 1× SP Conjugate was added to each well and incubated for 30 min before another round of washing. A 50 μL volume of Chromogen Substrate was added to each well and left to incubate under ambient light for approximately 25 min. Finally, 50 μL of stop solution was introduced into each well, resulting in a color change from blue to yellow. The absorbance of each well was scanned at 450 nm using Synergy H1 Hybrid Reader (Bio-Tek). After generating a standard curve, the unknown sample concentration was calculated from the Standard Curve.

### Oil Red O staining

Oil Red O (ORO) staining was conducted as previously described (Jing et al., 2023). In brief, O.C.T-embedded ovarian tissue sections (8 μm thickness) were taken out of the refrigerator to air-dry and fixed with 4% PFA for 20 min following washes with PBS for three times. Then, the sections were rinsed in 60% (*v*/*v*) isopropyl alcohol for 5 min. Without washing, the sections were promptly stained using 60% ORO solution for 11 min. To remove the background staining, the slides underwent a 10-s wash with 70% ethanol. Subsequently, the slides were rinsed and counterstained with Harris hematoxylin to visualize the nucleus once the color was suitable for microscopic observation before being mounted with 80% Glycerol/PBS. Images were captured with Olympus CKX41 microscope imaging system, and the ORO-positive areas were quantified with ImageJ.

### TUNEL staining

TUNEL staining was performed using the One Step TUNEL Apoptosis Assay Kit (Beyotime, C1088) following the manufacturer’s instruction to identify the apoptotic signals within ovarian tissues. Briefly, paraffin-embedded ovarian tissues sections (with a thickness of 5 μm) were routinely dewaxed to water. These sections were incubated with 20 μg/mL DNase-free proteinase K (dilution with 10 mmol/L Tris-HCl pH = 7.8) for 30 min at RT, followed by three rinses in PBS. Next, the sections were stained with TUNEL working solution at 37°C for 1 h. Then the slides were counterstained with Hoechst 33342 (Thermo Fisher, H3570, 1:1,000) to visualize the nucleus, and washed three times with PBS. Finally, the slides were mounted with VECTERSHIELD^®^ anti-fading mounting medium (Neobioscience, H-1000). Image capture was performed using the Zeiss LSM900 confocal system.

### 10× Visium raw data processing

The raw fastq data were aligned to Macaca_fascicularis_5.0 reference, and counted using spaceranger count (10× Visium, version 2.0.0) with the default parameters. The filtered matrixes were further analyzed with Giotto (version 2.0.2) (Dries et al., 2021). The “filterGiotto” function was used to filtered the raw data, after which 17,393 spots were retained. We used the following filtering criteria: “feat_det_in_min_cells = 50” and “expression_threshold = 1”.

### Sample integration, clustering, and identification of spot types

Data normalization, integration, dimensionality reduction, clustering, and differential gene expression analysis were performed under the corresponding pipeline of Giotto package (version 2.0.0) (Dries et al., 2021). The filtered count matrix of each sample was normalized using the “normalizeGiotto” function. The “calculateHVF” function was employed to compute highly variable features. The “runPCA” function was utilized for principal component analysis (PCA). Additionally, we used “runGiottoHarmony” function for data integration and “createNearestNetwork” function to create a nearest neighbor (NN) network based on default parameter. Clustering was performed using the “doLeidenCluster” function, which cluster cells using a NN-network and the Leiden community detection algorithm and run UMAP dimension reduction with “runUMAP” function. Next, spot types were annotated according to the expression levels of the canonical marker genes of germ cells or somatic cells. For spot type identification, the marker genes of each spot type were calculated using the “findMarkers_one_vs_all” function with the cutoff of Log_2_FC > 0.5 and FDR < 0.05. Marker genes for each spot type are shown in Table S1.

### Identification of aging-related DEGs

Aging-related DEG (aging DEG for abbreviation) analysis for each spot type between young and old groups was performed by “findScranMarkers” function of Giotto package. The aging-DEGs between two groups were filtered with the cutoff of |LogFC| > 0.5 and FDR < 0.05. DEGs for each spot type are shown in Table S2.

### Pathway enrichment analysis

The pathway enrichment analysis of aging DEGs and selected marker gene sets of each spot type were performed by Metascape (version 3.5) (Zhou et al., 2019) and ClusterProfiler (version 4.0.0) (Wu et al., 2021). For the latter tool, “compareCluster” function with the cutoff *q*-value < 0.05 was used. The results were further visualized with the “enrichplot” package (version 1.12.0) or ggplot2 R package.

### Gene set score analysis

Classical signaling pathway gene sets were downloaded from the KEGG (Kanehisa et al., 2021) and MSigDB (Liberzon et al., 2015) database. Aging-related gene set was obtained from Aging Atlas (Aging Atlas, 2021). A collection of gene sets associated with multiple ovarian diseases were downloaded from Harmonizome 3.0 (Rouillard et al., 2016). Fibrosis-related gene set was obtained from FibroAtlas (Liu et al., 2019). The gene set score of each spot was calculated using “AddModuleScore” function in Seurat package. In addition, we conducted intersection analysis for upregulated and downregulated DEGs with genes downloaded from the Aging Atlas, separately, to obtain gene sets of upregulated or downregulated OSAGs. The aging score was calculated by subtracting the OSAG-downregulated score from the OSAG-upregulated score. The analysis of score variations between the old group and the young group was conducted utilizing the ggpubr R package (version 0.6.0) through the application of the Wilcoxon test. Gene sets used in this study are shown in Table S3.

### Aging hotspot analysis

The Pearson correlation indexes of gene set scores between upregulated and downregulated OSAGs and 11 gene sets were calculated. Based on the values of correlation coefficient and significance, we selected pathways with the correlation coefficient *R* > 0.5 and the significance *P* values < 0.05, and named these pathways as primary contributors to ovarian aging (PCOA). Subsequently, we performed further analysis based on the selected pathway scores and aging scores. We labeled the spots with the highest 5% of each score as pathway/aging-sensitive spots and conducted an intersection analysis of these spots, ultimately obtaining aging hotspots for subsequent analysis.

### Pseudotime analysis

Pseudotime analysis was performed on TC, GC, and MT2^high^ spots with the Monocle2 R package (version 2.14.0) (Trapnell et al., 2014). The spot number of each spot type was down-sampled to 1,000. The trajectories were displayed in two-dimensional space using the DDRTree dimensionality reduction algorithm and then spots were ordered in pseudotime. The “BEAM_rest” functions of Monocle2 were used to identify DEGs along the pseudotime trajectory with the cutoff of *q*-value < 0.01.

### Spot-spot communication analysis

The potential interaction between different spot types was performed using the CellPhoneDB software (version 2.1.7) (Efremova et al., 2020). Only receptors and their ligands expressed in at least 10% spots of any spot types were considered as existing interactions. The threshold for valid spot–spot communication was *P*-value < 0.01. The spot–spot interaction pairs between MT2^high^ and other spots were calculated, and those pairs which lost and gained with age was further analyzed.

### Statistical analysis

All data were statistically analyzed using two-tailed Student’s *t*-test or Wilcoxon test to compare differences between young and old groups. The statistical analyses were performed using GraphPad Prism (version 9.5.1) and R packages. *P* values < 0.05 are considered statistically significant.

## Supplementary Material

pwad063_suppl_Supplementary_Figures_S1-S7

pwad063_suppl_Supplementary_Tables_S1

pwad063_suppl_Supplementary_Tables_S2

pwad063_suppl_Supplementary_Tables_S3

## Data Availability

The raw sequence data reported in this study have been deposited in the Genome Sequence Archive (GSA) with the accession number CRA013023.

## Code availability

All the custom codes are available from the corresponding authors upon reasonable request.

## References

[CIT0001] Adashi EY. Endocrinology of the ovary. Hum Reprod 1994;9:815–27. 7929728 10.1093/oxfordjournals.humrep.a138602

[CIT0002] Aging Atlas C. Aging Atlas: a multi-omics database for aging biology. Nucleic Acids Res 2021;49:D825–30. 33119753 10.1093/nar/gkaa894 PMC7779027

[CIT0003] Aging Biomarker C , Bao H, Cao J et al. Biomarkers of aging. Sci China Life Sci 2023;66:893–1066. 37076725 10.1007/s11427-023-2305-0 PMC10115486

[CIT0004] Ahmed TA , Ahmed SM, El-Gammal Z et al. Oocyte aging: the role of cellular and environmental factors and impact on female fertility. Adv Exp Med Biol 2020;1247:109–23. 31802446 10.1007/5584_2019_456

[CIT0005] Amargant F , Manuel SL, Tu Q et al. Ovarian stiffness increases with age in the mammalian ovary and depends on collagen and hyaluronan matrices. Aging Cell 2020;19:e13259. 33079460 10.1111/acel.13259 PMC7681059

[CIT0006] Ata B , Seyhan A, Seli E. Diminished ovarian reserve versus ovarian aging: overlaps and differences. Curr Opin Obstet Gynecol 2019;31:139–47. 30870184 10.1097/GCO.0000000000000536

[CIT0007] Auersperg N , Wong AS, Choi KC et al. Ovarian surface epithelium: biology, endocrinology, and pathology. Endocr Rev 2001;22:255–88. 11294827 10.1210/edrv.22.2.0422

[CIT0008] Bai H , Mu L, Qiu L et al. Complement C3 regulates inflammatory response and monocyte/macrophage phagocytosis of *Streptococcus agalactiae* in a teleost fish. Int J Mol Sci 2022;23:15586. 36555227 10.3390/ijms232415586 PMC9779060

[CIT0009] Ben Yaakov T , Wasserman T, Aknin E et al. Single-cell analysis of the aged ovarian immune system reveals a shift towards adaptive immunity and attenuated cell function. Elife 2023;12:e74915. 37096871 10.7554/eLife.74915 PMC10188116

[CIT0010] Birch J , Gil J. Senescence and the SASP: many therapeutic avenues. Genes Dev 2020;34:1565–76. 33262144 10.1101/gad.343129.120 PMC7706700

[CIT0011] Broekmans FJ , Soules MR, Fauser BC. Ovarian aging: mechanisms and clinical consequences. Endocr Rev 2009;30:465–93. 19589949 10.1210/er.2009-0006

[CIT0012] Brown HM , Russell DL. Blood and lymphatic vasculature in the ovary: development, function and disease. Hum Reprod Update 2014;20:29–39. 24097804 10.1093/humupd/dmt049

[CIT0013] Burris-Hiday SD , Scott EE. Steroidogenic cytochrome P450 17A1 structure and function. Mol Cell Endocrinol 2021;528:111261. 33781841 10.1016/j.mce.2021.111261 PMC8087655

[CIT0014] Cai Y , Song W, Li J et al. The landscape of aging. Sci China Life Sci 2022;65:2354–454. 36066811 10.1007/s11427-022-2161-3 PMC9446657

[CIT0015] Castrillon DH , Quade BJ, Wang TY et al. The human VASA gene is specifically expressed in the germ cell lineage. Proc Natl Acad Sci U S A 2000;97:9585–90. 10920202 10.1073/pnas.160274797 PMC16908

[CIT0016] Cheng C , Liu ZG. Autophagy and the metabolism of misfolding protein. Adv Exp Med Biol 2019;1206:375–420. 31776995 10.1007/978-981-15-0602-4_18

[CIT0017] Cheng S , Li Z, Gao R et al. A pan-cancer single-cell transcriptional atlas of tumor infiltrating myeloid cells. Cell 2021;184:792–809e23. 33545035 10.1016/j.cell.2021.01.010

[CIT0018] Debacq-Chainiaux F , Erusalimsky JD, Campisi J et al. Protocols to detect senescence-associated beta-galactosidase (SA-betagal) activity, a biomarker of senescent cells in culture and in vivo. Nat Protoc 2009;4:1798–806. 20010931 10.1038/nprot.2009.191

[CIT0019] Dries R , Zhu Q, Dong R et al. Giotto: a toolbox for integrative analysis and visualization of spatial expression data. Genome Biol 2021;22:78. 33685491 10.1186/s13059-021-02286-2 PMC7938609

[CIT0020] Duffy DM , Ko C, Jo M et al. Ovulation: parallels with inflammatory processes. Endocr Rev 2019;40:369–416. 30496379 10.1210/er.2018-00075 PMC6405411

[CIT0021] Efremova M , Vento-Tormo M, Teichmann SA et al. CellPhoneDB: inferring cell-cell communication from combined expression of multi-subunit ligand-receptor complexes. Nat Protoc 2020;15:1484–506. 32103204 10.1038/s41596-020-0292-x

[CIT0022] Fan X , Chuva de Sousa Lopes SM. Molecular makeup of the human adult ovary. Curr Opin Endocr Metab Res 2021;18:187–93.

[CIT0023] He S , Wang LH, Liu Y et al. Single-cell transcriptome profiling of an adult human cell atlas of 15 major organs. Genome Biol2020a;21:294. 33287869 10.1186/s13059-020-02210-0 PMC7720616

[CIT0024] He X , Memczak S, Qu J et al. Single-cell omics in ageing: a young and growing field. Nat Metab2020b;2:293–302. 32694606 10.1038/s42255-020-0196-7

[CIT0025] Huang D , Zuo Y, Zhang C et al. A single-nucleus transcriptomic atlas of primate testicular aging reveals exhaustion of the spermatogonial stem cell reservoir and loss of Sertoli cell homeostasis. Protein Cell 2022;14:887-907. 10.1093/procel/pwac057 PMC10691849 36929025

[CIT0026] Husain MA , Laurent B, Plourde M. APOE and Alzheimer’s disease: from lipid transport to physiopathology and therapeutics. Front Neurosci 2021;15:630502. 33679311 10.3389/fnins.2021.630502 PMC7925634

[CIT0027] Isola JVV , Ocañas SR, Hubbart CR et al. A single-cell atlas of the aging murine ovary. bioRxiv 2023.

[CIT0028] Jia L , Wang W, Liang J et al. Analyzing the cellular and molecular atlas of ovarian mesenchymal cells provides a strategy against female reproductive aging. Sci China Life Sci 2023;66:2818–2836. 37460714 10.1007/s11427-022-2335-6

[CIT0029] Jing Y , Zuo Y, Yu Y et al. Single-nucleus profiling unveils a geroprotective role of the FOXO3 in primate skeletal muscle aging. Protein Cell 2023;14:497–512. 36921027 10.1093/procel/pwac061 PMC10305740

[CIT0030] Kanehisa M , Furumichi M, Sato Y et al. KEGG: integrating viruses and cellular organisms. Nucleic Acids Res 2021;49:D545–51. 33125081 10.1093/nar/gkaa970 PMC7779016

[CIT0031] Komatsu K , Masubuchi S. Observation of the dynamics of follicular development in the ovary. Reprod Med Biol 2017;16:21–7. 29259446 10.1002/rmb2.12010 PMC5715870

[CIT0032] Lehallier B , Gate D, Schaum N et al. Undulating changes in human plasma proteome profiles across the lifespan. Nat Med 2019;25:1843–50. 31806903 10.1038/s41591-019-0673-2 PMC7062043

[CIT0033] Leng SX , Pawelec G. Single-cell immune atlas for human aging and frailty. Life Med 2022;1:67–70. 36699943 10.1093/lifemedi/lnac013 PMC9869752

[CIT0034] Lengyel E , Li Y, Weigert M et al. A molecular atlas of the human postmenopausal fallopian tube and ovary from single-cell RNA and ATAC sequencing. Cell Rep 2022;41:111838. 36543131 10.1016/j.celrep.2022.111838 PMC11295111

[CIT0035] Li J , Zheng Y, Yan P et al. A single-cell transcriptomic atlas of primate pancreatic islet aging. Natl Sci Rev 2021;8:nwaa127. 34691567 10.1093/nsr/nwaa127 PMC8288398

[CIT0036] Liberzon A , Birger C, Thorvaldsdóttir H et al. The Molecular Signatures Database (MSigDB) hallmark gene set collection. Cell Syst 2015;1:417–25. 26771021 10.1016/j.cels.2015.12.004 PMC4707969

[CIT0037] Lin SN , Musso A, Wang J et al. Human intestinal myofibroblasts deposited collagen VI enhances adhesiveness for T cells—a novel mechanism for maintenance of intestinal inflammation. Matrix Biol 2022;113:1–21. 36108990 10.1016/j.matbio.2022.09.001 PMC10043923

[CIT0038] Liu J , Sun D, Liu J et al. FibroAtlas: a database for the exploration of fibrotic diseases and their genes. Cardiol Res Pract 2019;2019:4237285. 32082621 10.1155/2019/4237285 PMC7012261

[CIT0039] López-Otín C , Blasco MA, Partridge L et al. Hallmarks of aging: an expanding universe. Cell 2023;186:243–78. 36599349 10.1016/j.cell.2022.11.001

[CIT0040] Lovell TM , Gladwell RT, Groome NP et al. Ovarian follicle development in the laying hen is accompanied by divergent changes in inhibin A, inhibin B, activin A and follistatin production in granulosa and theca layers. J Endocrinol 2003;177:45–55. 12697036 10.1677/joe.0.1770045

[CIT0041] Ma S , Sun S, Geng L et al. Caloric restriction reprograms the single-cell transcriptional landscape of Rattus Norvegicus aging. Cell 2020;180:984–1001.e22. 32109414 10.1016/j.cell.2020.02.008

[CIT0042] Ma S , Sun S, Li J et al. Single-cell transcriptomic atlas of primate cardiopulmonary aging. Cell Res 2021;31:415–32. 32913304 10.1038/s41422-020-00412-6 PMC7483052

[CIT0043] Ma S , Chi X, Cai Y et al. Decoding aging hallmarks at the single-cell level. Annu Rev Biomed Data Sci 2023;6:129–52. 37127051 10.1146/annurev-biodatasci-020722-120642

[CIT0044] Machlin JH , Shikanov A. Single-cell RNA-sequencing of retrieved human oocytes and eggs in clinical practice and for human ovarian cell atlasing. Mol Reprod Dev 2022;89:597–607. 36264989 10.1002/mrd.23648 PMC9805491

[CIT0045] Mara JN , Zhou LT, Larmore M et al. Ovulation and ovarian wound healing are impaired with advanced reproductive age. Aging (Albany NY) 2020;12:9686–713. 32407290 10.18632/aging.103237 PMC7288922

[CIT0046] Mishina T , Tabata N, Hayashi T et al. Single-oocyte transcriptome analysis reveals aging-associated effects influenced by life stage and calorie restriction. Aging Cell 2021;20:e13428. 34245092 10.1111/acel.13428 PMC8373347

[CIT0047] Pal L , Santoro N. Premature ovarian failure (POF): discordance between somatic and reproductive aging. Ageing Res Rev 2002;1:413–23. 12067595 10.1016/s1568-1637(02)00009-0

[CIT0048] Perry JR , Murray A, Day FR et al. Molecular insights into the aetiology of female reproductive ageing. Nat Rev Endocrinol 2015;11:725–34. 26460341 10.1038/nrendo.2015.167 PMC6309261

[CIT0049] Petrosino JM , Disilvestro D, Ziouzenkova O. Aldehyde dehydrogenase 1A1: friend or foe to female metabolism? Nutrients 2014;6:950–73. 24594504 10.3390/nu6030950 PMC3967171

[CIT0050] Read A , Schröder M. The unfolded protein response: an overview. Biology (Basel) 2021;10:384. 33946669 10.3390/biology10050384 PMC8146082

[CIT0051] Rouillard AD , Gundersen GW, Fernandez NF et al. The harmonizome: a collection of processed datasets gathered to serve and mine knowledge about genes and proteins. Database (Oxford) 2016;2016:baw100. 27374120 10.1093/database/baw100 PMC4930834

[CIT0052] Sasano H , Suzuki T. Localization of steroidogenesis and steroid receptors in human corpus luteum classification of human corpus luteum (CL) into estrogen-producing degenerating CL, and nonsteroid-producing degenerating CL. Semin Reprod Endocrinol 1997;15:345–51. 9580943 10.1055/s-2008-1068372

[CIT0053] Shi Y , Guo Y, Zhou J et al. A spatiotemporal gene expression and cell atlases of the developing rat ovary. Cell Prolif 2023;56:e13516. doi:10.1111/cpr.13516 37309718 PMC10693188

[CIT0054] Sun Y , Li Q, Kirkland JL. Targeting senescent cells for a healthier longevity: the roadmap for an era of global aging. Life Med 2022;1:103–19. 36699942 10.1093/lifemedi/lnac030 PMC9869767

[CIT0055] Sun G , Zheng Y, Fu X et al. Single-cell transcriptomic atlas of mouse cochlear aging. Protein Cell 2023;14:180–201. 36933008 10.1093/procel/pwac058 PMC10098046

[CIT0056] Tang F , Barbacioru C, Wang Y et al. mRNA-Seq whole-transcriptome analysis of a single cell. Nat Methods 2009;6:377–82. 19349980 10.1038/nmeth.1315

[CIT0057] Tew WP. Ovarian cancer in the older woman. J Geriatr Oncol 2016;7:354–61. 27499341 10.1016/j.jgo.2016.07.008

[CIT0058] Trapnell C , Cacchiarelli D, Grimsby J et al. The dynamics and regulators of cell fate decisions are revealed by pseudotemporal ordering of single cells. Nat Biotechnol 2014;32:381–6. 24658644 10.1038/nbt.2859 PMC4122333

[CIT0059] Vollenhoven B , Hunt S. Ovarian ageing and the impact on female fertility. F1000Res 2018;7:F1000 Faculty Rev-1835. 10.12688/f1000research.16509.1 PMC6259486 30542611

[CIT0060] Wagner M , Yoshihara M, Douagi I et al. Single-cell analysis of human ovarian cortex identifies distinct cell populations but no oogonial stem cells. Nat Commun 2020;11:1147. 32123174 10.1038/s41467-020-14936-3 PMC7052271

[CIT0061] Wang S , Hu B, Ding Z et al. ATF6 safeguards organelle homeostasis and cellular aging in human mesenchymal stem cells. Cell Discov 2018;4:2. 29423270 10.1038/s41421-017-0003-0 PMC5798892

[CIT0062] Wang S , Zheng Y, Li J et al. Single-cell transcriptomic atlas of primate ovarian aging. Cell 2020;180:585–600 e519. 32004457 10.1016/j.cell.2020.01.009

[CIT0063] Wang S , Cheng F, Ji Q et al. Hyperthermia differentially affects specific human stem cells and their differentiated derivatives. Protein Cell 2022;13:615–22. 34719769 10.1007/s13238-021-00887-y PMC9232687

[CIT0064] Wei Y , Yu R, Cheng S et al. Single-cell profiling of mouse and primate ovaries identifies high levels of EGFR for stromal cells in ovarian aging. Mol Ther Nucleic Acids 2023;31:1–12. 36570672 10.1016/j.omtn.2022.11.020 PMC9761475

[CIT0065] Woad KJ , Robinson RS. Luteal angiogenesis and its control. Theriogenology 2016;86:221–8. 27177965 10.1016/j.theriogenology.2016.04.035

[CIT0066] Wu T , Hu E, Xu S et al. clusterProfiler 40: a universal enrichment tool for interpreting omics data. Innovation (Camb) 2021;2:100141. 34557778 10.1016/j.xinn.2021.100141 PMC8454663

[CIT0067] Wu J , Liu Y, Song Y et al. Aging conundrum: a perspective for ovarian aging. Front Endocrinol (Lausanne) 2022;13:952471. 36060963 10.3389/fendo.2022.952471 PMC9437485

[CIT0068] Yuan L , Yin P, Yan H et al. Single-cell transcriptome analysis of human oocyte ageing. J Cell Mol Med 2021;25:6289–303. 34037315 10.1111/jcmm.16594 PMC8256362

[CIT0069] Zhang W , Zhang S, Yan P et al. A single-cell transcriptomic landscape of primate arterial aging. Nat Commun 2020;11:2202. 32371953 10.1038/s41467-020-15997-0 PMC7200799

[CIT0070] Zhang B , Yan H, Liu X et al. SenoIndex: S100A8/S100A9 as a novel aging biomarker. Life Med 2023;2:lnad022. 10.1093/lifemedi/lnad022 PMC11749476 39872551

[CIT0071] Zhao H , Ji Q, Wu Z et al. Destabilizing heterochromatin by APOE mediates senescence. Nat Aging 2022;2:303–16. 37117743 10.1038/s43587-022-00186-z

[CIT0072] Zhou Y , Zhou B, Pache L et al. Metascape provides a biologist-oriented resource for the analysis of systems-level datasets. Nat Commun 2019;10:1523. 30944313 10.1038/s41467-019-09234-6 PMC6447622

[CIT0073] Zhou T , Kiran M, Lui KO et al. Decoding liver fibrogenesis with single-cell technologies. Life Med 2022;1:333–44. 10.1093/lifemedi/lnac040 PMC11749458 39872749

[CIT0074] Zhu Z , Xu W, Liu L. Ovarian aging: mechanisms and intervention strategies. Med Rev (Berlin, Germany) 2022;2:590–610. 10.1515/mr-2022-0031 PMC10471094 37724254

