# Supplementary material for: Aging hallmarks of the primate ovary revealed by spatiotemporal transcriptomics
Source: Protein Cell. 2023 Dec 21;15(5):364–84. doi: 10.1093/procel/pwad063 (PMC11075111; doi:10.1093/procel/pwad063)
Supplement: pwad063_suppl_Supplementary_Figures_S1-S7 [file pwad063_suppl_supplementary_figures_s1-s7.pdf]

Supplemental Materials

Figure S1. Quality control for spatial transcriptome data of NHP ovarian aging.

(A) H&E staining (top) and spot type annotation of spatial transcriptomic data (bottom) for all samples in this study.
(B) Spatial visualization showing the number of genes detected in each spot of all samples.
(C) Bar plot showing the number of genes detected in each spot of all samples.
(D) Bar plot showing the mean reads of each spot for all samples.
(E) Bar plot showing the spot number for all samples.

Figure S2. Spatial expression of marker genes for major spot types in monkey ovary.

Figure S3. CD31 immunofluorescence staining of ovaries from young and old monkeys.
Representative images are shown on the left. Scale bars, 20 μm and 10 μm (zoomed-in images). The numbers of CD31-positive cells and CD31-positive area were quantified as fold changes (Old vs. Young), and presented as mean ± SEMs on the right. n = 4 monkeys for each group.

Figure S4. Age-related changes of a panel of aging hallmarks in NHP ovaries.
(A) CD163 immunofluorescence staining of ovaries from young and old monkeys. Representative images are shown on the left. Scale bars, 20 μm and 10 μm (zoomed-in images). The numbers of CD163-positive cells were quantified as fold changes (Old vs. Young), and presented as mean ± SEMs on the right. n = 4 monkeys for each group.
(B) Violin plot showing the gene set score of inflammatory response-related genes across different spot types in young and old groups.
(C) Violin plot showing the gene set score of SASP-related genes across different spot types in young and old groups.
(D) Violin plot showing the gene set score of fibrosis-related genes across different spot types in young and old groups.
(E) Ridge plots (left) and violin plot (right) showing the gene set score of TGF-β pathway-related genes in young and old groups. The corresponding dashed lines represent the median of each group.
(F) Violin plot showing the gene set score of lipid storage-related genes across different spot types in young and old groups.
(G) Ridge plots (left) and violin plot (right) showing the gene set score of ATF6 pathway-related genes in young and old groups. The corresponding dashed lines represent the median of each group.
(H) Ridge plots (left) and violin plot (right) showing the global distribution density the gene set score of IRE1 pathway-related genes in young and old groups. The corresponding dashed lines represent the median of each group.
(I) Ridge plots (left) and violin plot (right) showing the gene set score of PERK pathway-related genes in young and old groups. The corresponding dashed lines represent the median of each group.

(J) Ridge plots (left) and violin plot (right) showing the gene set score of autophagy-related genes in young and old groups. The corresponding dashed lines represent the median of each group.
(K) Violin plot showing the gene set score of apoptosis-related genes across different spot types in young and old groups.
(L) Cleaved-caspase 3 immunostaining of ovarian tissues from young and aged monkeys. Representative images are shown on the left. Scale bar, 20 μm and 10 μm (zoomed-in images). Cleaved-caspase 3-positive cells in the tissues were quantified as fold changes (Old vs. Young), and shown as mean ± SEMs on the right. n = 4 monkeys for each group.

Figure S5. Analysis of the canonical aging hallmark-related genes set in NHP ovaries.
(A) Violin plot showing the gene set score of ROS-related genes across different spot types in young and old groups.
(B) Violin plot showing the gene set score of DNA repair-related genes across different spot types in young and old groups.
(C) Violin plot showing the gene set score of senescence-related genes across different spot types in young and old groups.
(D) SA-β-Gal staining of ovaries from young and old monkeys. Scale bars, 200 μm and 50 μm (zoomed-in images). SA-β-Gal-positive areas were quantified as fold changes (Old vs. Young), and presented as mean ± SEMs on the right. n = 4 monkeys for each group.

Figure S6. Correlation analysis between OSAGs score and multiple major aging-related pathways.
(A) The scatter plot showing the correlation coefficients between OSAG-upregulated or OSAG-downregulated gene set scores and gene set scores of 11 age-related pathways.
(B) Correlation analysis between OSAG-upregulated set score and gene set scores of 11 age-related pathways across different spot types in monkey ovary.
(C) The spatial distribution and colocalization presentation of OSAG-upregulated gene set score and scores of fibrosis, senescence, and inflammatory response-related gene sets.

Figure S7. The integrative analysis of APOE-positive spots and spots with a high PCOA score.
(A) The spatial distribution and colocalization presentation of spots highly expressing *APOE* and those with a high PCOA score, including SASP-related gene set score, fibrosis-related gene set score, senescence-related gene set score and inflammatory response-related gene set score.
(B) The scatter plot showing the correlation coefficient between *MT2* expression and *APOE* expression.
(C) The spatial distribution and colocalization presentation of *MT2* expression and *APOE* expression in cynomolgus monkey ovary.

Supplementary Tables

Table S1. Marker genes for each spot type in non-human primate ovaries.

Table S2. Aging -related differentially expressed genes (DEGs) for each spot type.
Table S3. Gene list for genes used in this study.

Figure S1

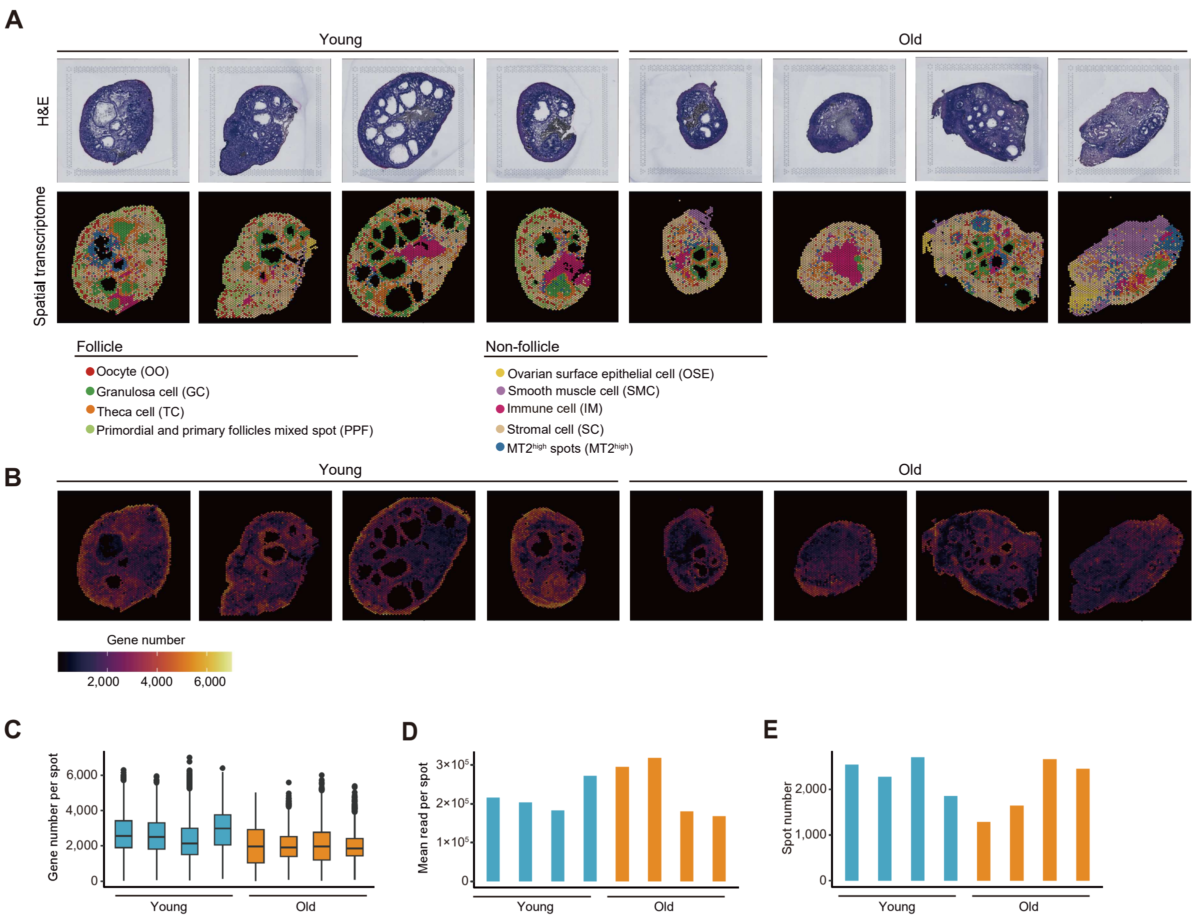
A
Young
Old
H&E
Spatial transcriptome
Follicle
Oocyte (OO)
Granulosa cell (GC)
Theca cell (TC)
Primordial and primary follicles mixed spot (PPF)
Non-follicle
Ovarian surface epithelial cell (OSE)
Smooth muscle cell (SMC)
Immune cell (IM)
Stromal cell (SC)
MT2high spots (MT2high)
B
Young
Old
Gene number
2,000
4,000
6,000
C
Gene number per spot
0
2,000
4,000
6,000
Young
Old
D
Mean read per spot
0
1×10⁵
2×10⁵
3×10⁵
Young
Old
E
Spot number
0
1,000
2,000
Young
Old

Figure S2

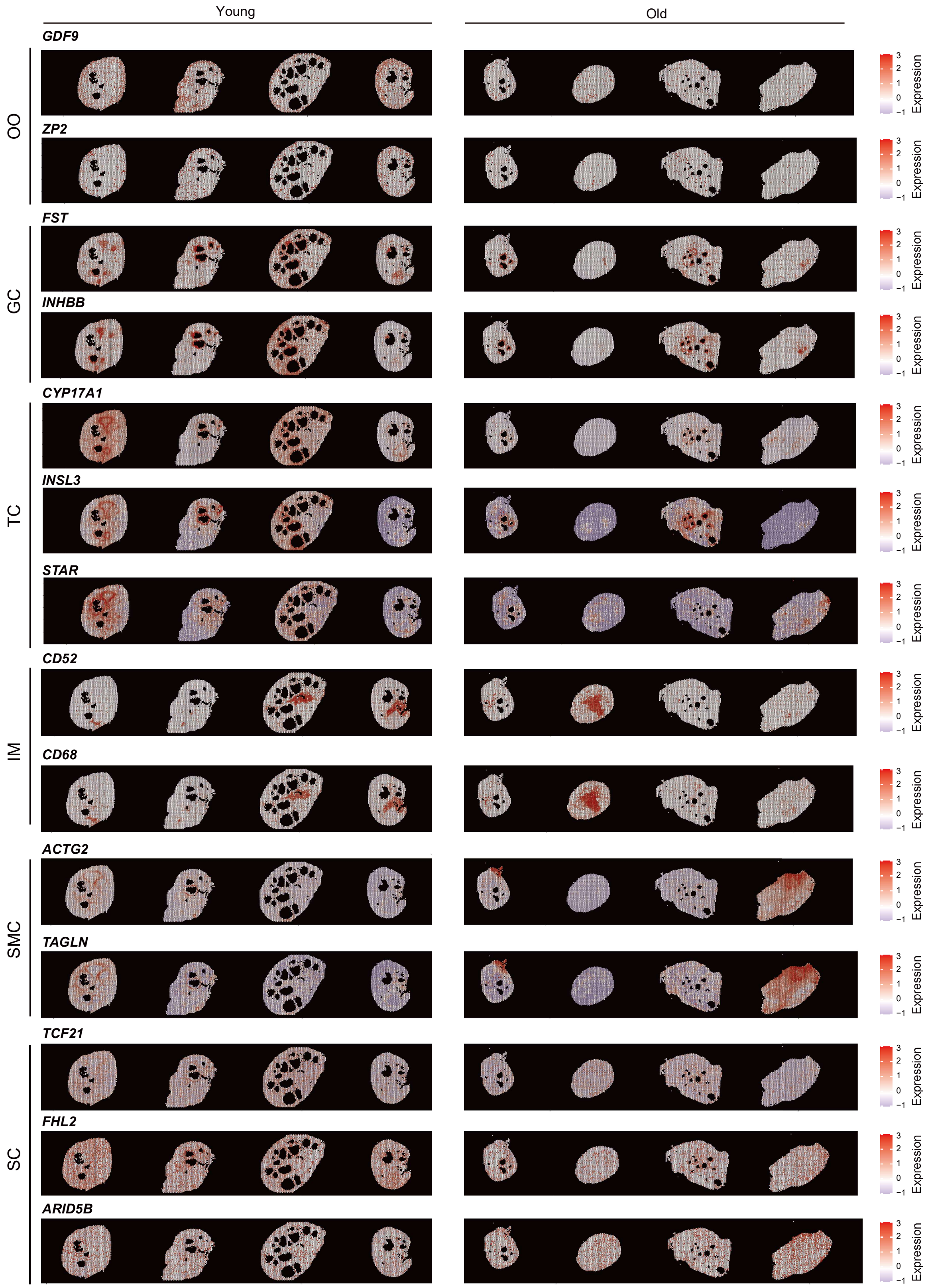

Young
Old
GDF9
ZP2
FST
INHBB
CYP17A1
INSL3
STAR
CD52
CD68
ACTG2
TAGLN
TCF21
FHL2
ARID5B
OO
GC
TC
IM
SMC
SC
Expression

Figure S3

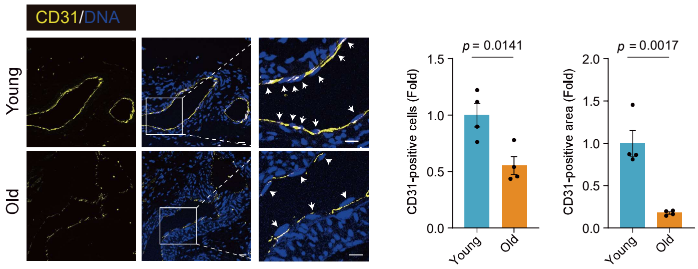
CD31/DNA
Young
Old
p = 0.0141
CD31-positive cells (Fold)
1.5
1.0
0.5
0.0
Young
Old
p = 0.0017
CD31-positive area (Fold)
2.0
1.5
1.0
0.5
0.0
Young
Old

Figure S4

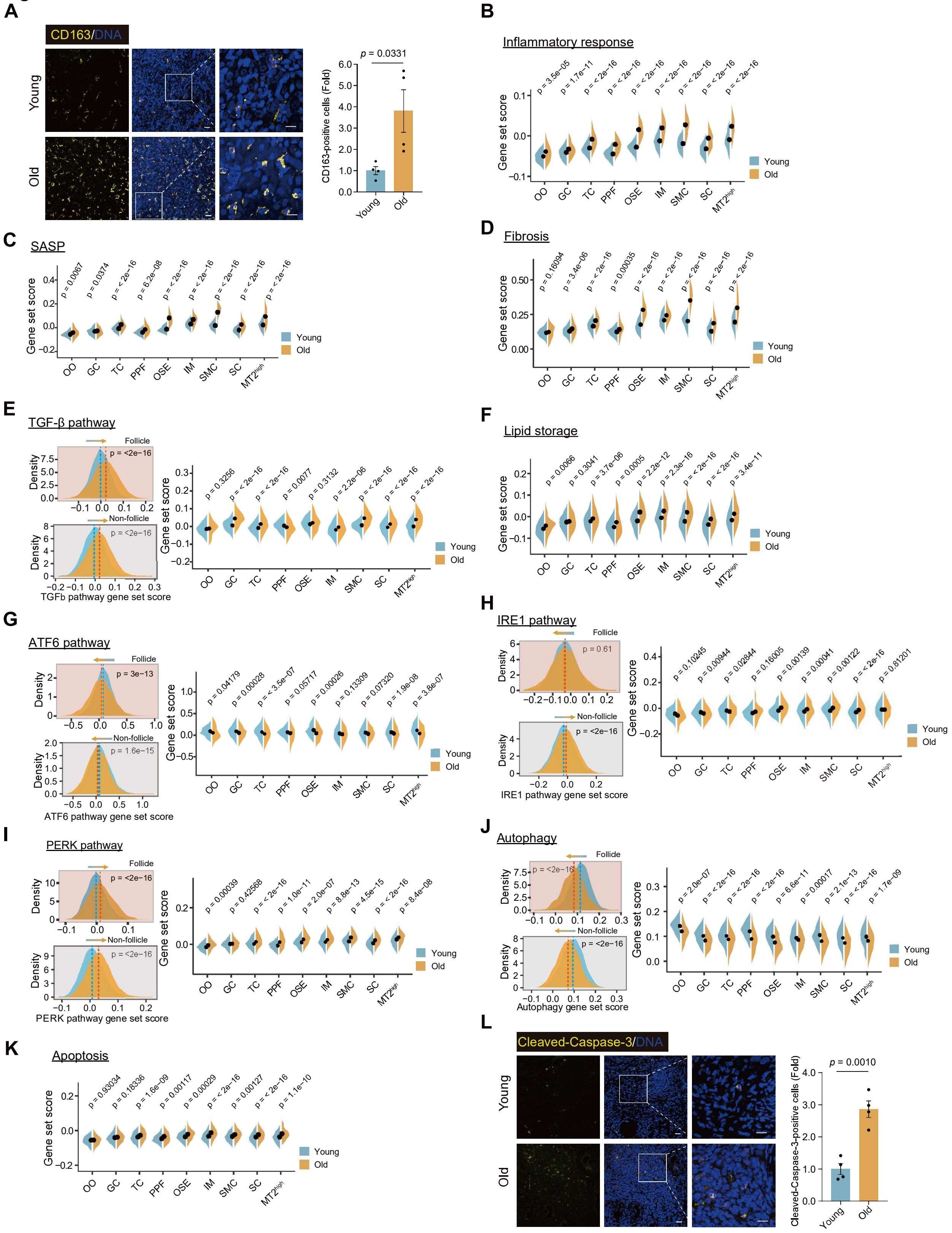
A
CD163/DNA
Young
Old
p = 0.0331
CD163-positive cells (Fold)
B
Inflammatory response
Gene set score
Young
Old
C
SASP
D
Fibrosis
E
TGF-β pathway
Follicle
Non-follicle
Density
TGFb pathway gene set score
F
Lipid storage
G
ATF6 pathway
ATF6 pathway gene set score
H
IRE1 pathway
IRE1 pathway gene set score
I
PERK pathway
PERK pathway gene set score
J
Autophagy
Autophagy gene set score
K
Apoptosis
L
Cleaved-Caspase-3/DNA
p = 0.0010
Cleaved-Caspase-3-positive cells (Fold)
OO
GC
TC
PPF
OSE
IM
SMC
SC
MT2high

Figure S5

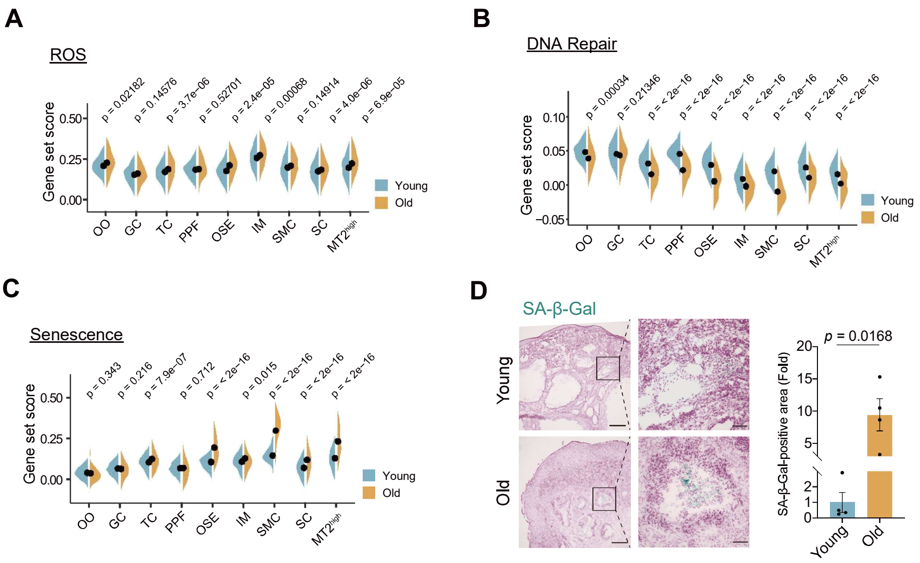
A
ROS
Gene set score
p = 0.02182
p = 0.14576
p = 3.7e−06
p = 0.52701
p = 2.4e−05
p = 0.00068
p = 0.14914
p = 4.0e−06
p = 6.9e−05
OO GC TC PPF OSE IM SMC SC MT2high
Young
Old
B
DNA Repair
Gene set score
p = 0.00034
p = 0.21346
p = < 2e−16
OO GC TC PPF OSE IM SMC SC MT2high
Young
Old
C
Senescence
Gene set score
p = 0.343
p = 0.216
p = 7.9e−07
p = 0.712
p = < 2e−16
p = 0.015
OO GC TC PPF OSE IM SMC SC MT2high
Young
Old
D
SA-β-Gal
Young
Old
p = 0.0168
SA-β-Gal-positive area (Fold)
Young Old

Figure S6

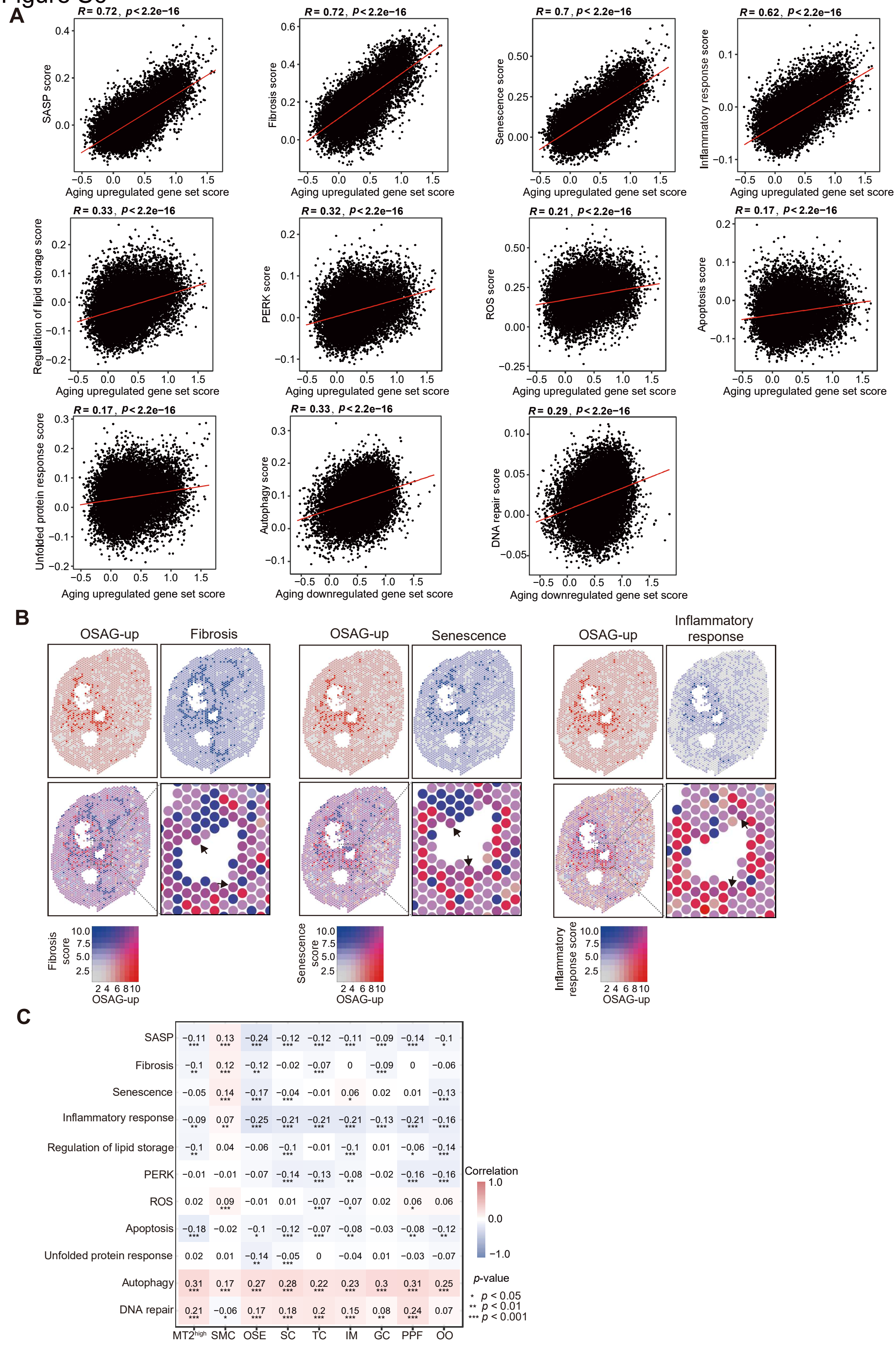
A
R = 0.72, p < 2.2e−16
SASP score
R = 0.72, p < 2.2e−16
Fibrosis score
R = 0.7, p < 2.2e−16
Senescence score
R = 0.62, p < 2.2e−16
Inflammatory response score
R = 0.33, p < 2.2e−16
Regulation of lipid storage score
R = 0.32, p < 2.2e−16
PERK score
R = 0.21, p < 2.2e−16
ROS score
R = 0.17, p < 2.2e−16
Apoptosis score
R = 0.17, p < 2.2e−16
Unfolded protein response score
R = 0.33, p < 2.2e−16
Autophagy score
R = 0.29, p < 2.2e−16
DNA repair score
Aging upregulated gene set score
Aging downregulated gene set score
B
OSAG-up
Fibrosis
Senescence
Inflammatory response
Fibrosis score
Senescence score
Inflammatory response score
OSAG-up
C
SASP −0.11 0.13 −0.24 −0.12 −0.12 −0.11 −0.09 −0.14 −0.1
Fibrosis −0.1 0.12 −0.12 −0.02 −0.07 0 −0.09 0 −0.06
Senescence −0.05 0.14 −0.17 −0.04 −0.01 0.06 0.02 0.01 −0.13
Inflammatory response −0.09 0.07 −0.25 −0.21 −0.21 −0.21 −0.13 −0.21 −0.16
Regulation of lipid storage −0.1 0.04 −0.06 −0.1 −0.01 −0.1 0.01 −0.06 −0.14
PERK −0.01 −0.01 −0.07 −0.14 −0.13 −0.08 −0.02 −0.16 −0.16
ROS 0.02 0.09 −0.01 0.01 −0.07 −0.07 0.02 0.06 0.06
Apoptosis −0.18 −0.02 −0.1 −0.12 −0.07 −0.08 −0.03 −0.08 −0.12
Unfolded protein response 0.02 0.01 −0.14 −0.05 0 −0.04 0.01 −0.03 −0.07
Autophagy 0.31 0.17 0.27 0.28 0.22 0.23 0.3 0.31 0.25
DNA repair 0.21 −0.06 0.17 0.18 0.2 0.15 0.08 0.24 0.07
MT2high SMC OSE SC TC IM GC PPF OO
Correlation
1.0
0.0
−1.0
p-value
* p < 0.05
** p < 0.01
*** p < 0.001

Figure S7

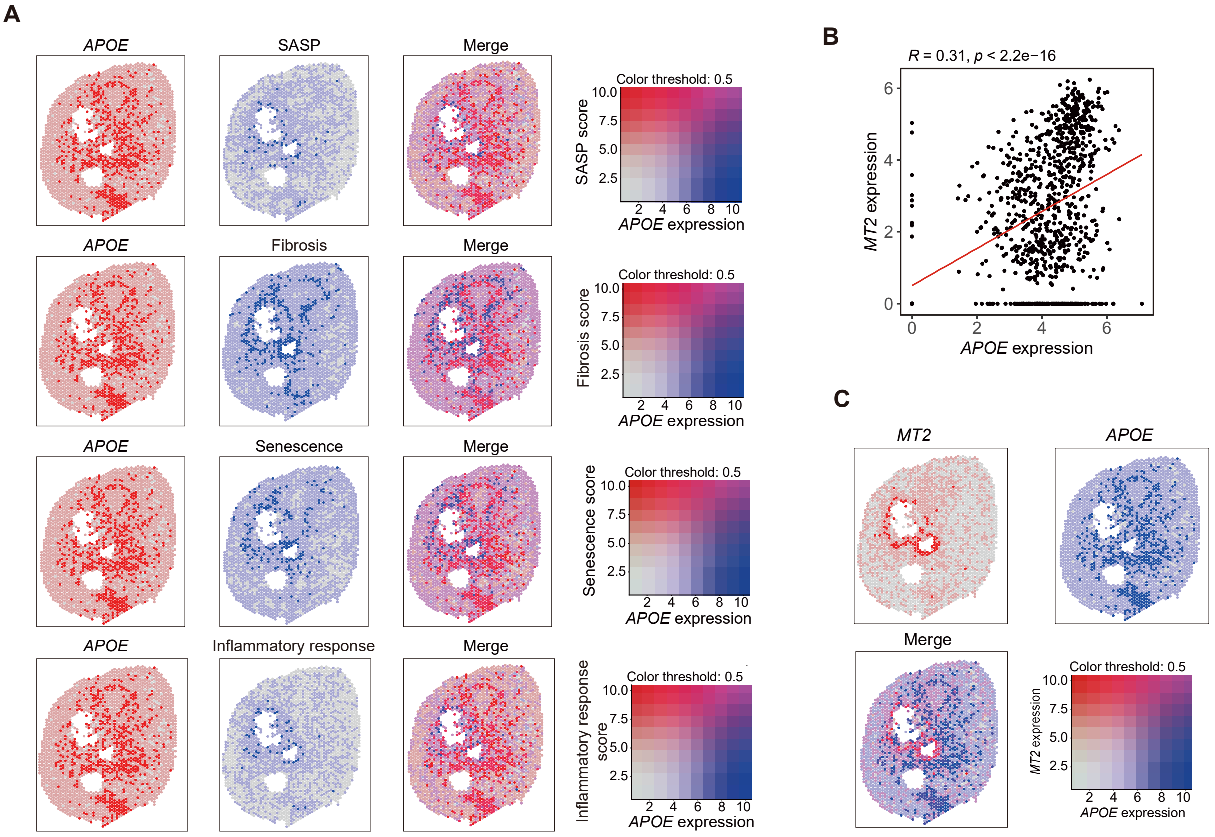
A
APOE
SASP
Merge
Color threshold: 0.5
SASP score
APOE expression
Fibrosis
Fibrosis score
Senescence
Senescence score
Inflammatory response
Inflammatory response score
B
R = 0.31, p < 2.2e−16
MT2 expression
APOE expression
C
MT2
APOE
Merge
Color threshold: 0.5
MT2 expression
APOE expression
